# Comparative genomic analyses of *Escherichia coli* ST405 strains from Pakistan

**DOI:** 10.1128/msystems.01685-25

**Published:** 2026-03-16

**Authors:** Nam-Hoon Kim, Jung Hun Lee, Saba Yasmin, Abdul Rauf Tareen, Kyung-Min Jang, Chanyeong Jeong, Byeonghyeon Kang, Gwangje Lee, Dong-Won Lee, Rabaab Zahra, Dae-Wi Kim, Sang Hee Lee

**Affiliations:** 1 Department of Life Sciences, Jeonbuk National University 26714, Jeonju, South Korea; 2 Research Institute for Materials and Energy Sciences, Jeonbuk National University 26714, Jeonju, South Korea; 3 National Leading Research Laboratory of Drug Resistance Proteomics, Department of Biological Sciences, Myongji University 65463, Yongin, South Korea; 4 Department of Microbiology, Quaid-i-Azam University 66757, Islamabad, Pakistan; 5 Department of Clinical Microbiology, Trinity College Dublin, St James's Hospital 8809, Dublin, Ireland; University of Technology Sydney, Sydney, NSW, Australia

**Keywords:** high-risk clone, ST405, comparative genomics, *bla*_NDM-5_, plasmid, IS*26*

## Abstract

*Escherichia coli* sequence type (ST) 405, a high-risk international clone, has emerged as a critical lineage for the dissemination of antibiotic resistance genes (ARGs), including the *bla*_NDM-5_ gene. This study aims to elucidate the genomic landscape of ST405 through comparative genomic analysis of both publicly available ST405 genomes and three ST405 isolates from sewage and wild-bird fecal samples in Pakistan. A total of 1,778 *E. coli* ST405 genomes were investigated through phylogenomic and population-structure analyses, revealing distinct clusters and subgroup lineages within this ST. Among these, subgroup B was identified as a pivotal contributor to the dissemination of the *bla*_NDM-5_ gene within ST405. Temporal analysis indicates that subgroup B is expanding over time, raising concerns regarding its potential contributions to increased antibiotic resistance within ST405 populations. Genetic-structure analyses of *bla*_NDM-5_-carrying plasmids of the sewage isolates uncovered the presence of nearly identical plasmids in other multidrug-resistant STs, ST156 and ST648, suggesting a genetic linkage among these STs. Additionally, a novel *bla*_NDM-5_ genetic context was identified in the animal isolate, characterized by unique composite-transposon and inversion structures mediated by IS*26* replicative transposition, highlighting the dynamic evolution of the ST405-associated plasmid. These findings underscore the intra-ST dissemination of the *bla*_NDM-5_ gene and the ongoing diversification of its genetic context within ST405. This comprehensive analysis advances our understanding of ST405, providing insights into the genetic mechanisms underlying its enhanced antibiotic resistance and the emergence of a novel ARG-carrying plasmid within the One Health framework.

**IMPORTANCE:**

This study provides a comprehensive genomic landscape of ST405, a high-risk international clone and a carrier of the *bla*_NDM-5_ gene, revealing phylogenetically distinct patterns in the distribution of antibiotic resistance genes and virulence factors, and a critical phylogenetic lineage that serves as a primary reservoir of the *bla*_NDM-5_ gene. Furthermore, the genetic linkage between ST405 and other STs (ST156 and ST648) through the sharing of identical *bla*_NDM-5_-carrying plasmids, and the emergence of a novel *bla*_NDM-5_ genetic structure in an animal isolate, underscore the pivotal role of ST405 in the dissemination of the *bla*_NDM-5_ gene. These findings highlight the public health significance of ST405 and its contribution to the global spread of carbapenem resistance.

## INTRODUCTION

*Escherichia coli* is recognized as a commensal bacterium that colonizes the gastrointestinal (GI) tract of vertebrates. However, certain strains exhibit pathogenic potential, contributing to a wide range of infectious diseases in their host (1). Based on the site of infection, pathogenic *E. coli* can be broadly categorized into intraintestinal pathogenic *E. coli* (InPEC) and extraintestinal pathogenic *E. coli* (ExPEC) (2). InPEC group includes several well-characterized pathotypes, such as enteropathogenic *E. coli*, enterohemorrhagic *E. coli*, enterotoxigenic *E. coli*, enteroaggregative *E. coli*, enteroaggregative hemorrhagic *E. coli*, and adherent-invasive *E. coli*. In contrast, ExPEC strains cause infections beyond the intestinal tract and include uropathogenic *E. coli*, neonatal meningitis-associated *E. coli*, and avian pathogenic *E. coli* (2). The pathogenicity of these *E. coli* strains is largely determined by their virulence factors (VFs), which facilitate colonization, immune evasion, and host-tissue invasion. These VFs can be acquired and disseminated through horizontal gene transfer (HGT), primarily mediated by mobile genetic elements (MGEs) (3, 4). The ability of *E. coli* to acquire new virulence determinants through HGT underscores its adaptability and potential to cause severe infections across diverse hosts and environments.

*E. coli* strains carrying antibiotic resistance genes (ARGs) pose a significant global health concern, as they have been isolated not only from infected patients but also from the gut microbiota of healthy humans (5). The acquisition of ARGs via various MGEs has contributed to the emergence of multidrug-resistant and extensively drug-resistant *E. coli*, complicating treatment options and increasing the burden of the resistance. Given the high incidence of *E. coli*-associated infections, the horizontal transfer of ARGs remains one of the most pressing challenges in public health (6, 7). Among the ARGs of greatest concern are those encoding carbapenemases and cephalosporinases, which confer resistance to β-lactam antibiotics, a critical class of antimicrobial agents. Notably, the prevalence of *E. coli* strains harboring NDM family β-lactamases (carbapenemases) has been rising annually in Europe, with increasing reports of strains exhibiting resistance to last-line antimicrobial therapies (8). Additionally, extended-spectrum β-lactamase (ESBL; cephalosporinase)-encoding genes, such as CTX-M family β-lactamases, are frequently detected in pathogenic *E. coli* strains (9). A previous study identified sequence type (ST) 167, ST410, ST405, and ST361 as major carriers of the *bla*_NDM-5_ gene (8). For *bla*_CTX-M-15_, it has been announced that the gene has been specifically associated with ST405 (10). ST405, classified as a high-risk clone, is widely distributed across humans, animals, and the environment, further underscoring its significance in ARG dissemination (11). A recent study reported the phenotypic and genomic characteristics of 26 carbapenem-resistant *E. coli* ST405 strains isolated from a tertiary hospital over an 8 year period. The authors also conducted comparative genomics analyses of this ST using 2,062 ST405 strains from the EnteroBase database, providing the understanding of the classification of phylogenomic clades, FimH types, *bla*_NDM_ types, and *bla*_CTX-M_ types (12). However, despite these studies, a comprehensive understanding of the ST405 genomic landscape remains incomplete. In particular, the interplay between resistance genes, plasmid structures, and mobile genetic elements across a globally representative data set requires further investigation. In this context, comparative genomics of ST405 provides a framework to characterize its global population structure and the dissemination patterns of clinically important ARGs, including *bla*_NDM-5_ (13, 14).

In this study, *E. coli* strains were isolated from sewage and wild-bird feces in Pakistan, and their complete genome sequences were obtained. Among these, three strains were identified as ST405, a high-risk clone associated with antibiotic resistance and human infections. Although ST405 is predominantly linked to clinical cases, its detection in environmental and animal sources underscores the necessity for further investigation into its ecological distribution and evolutionary adaptations. Despite its strong association with major VFs and ARGs, comparative genomic studies on ST405 remain limited. This study conducted a comprehensive comparative genomic analysis of ST405, integrating data from environmental and animal isolates alongside publicly available ST405 genomes. The analysis focused on characterizing the genomic landscape of this high-risk international clone, including its phylogenomic clades, One Health (human, animal, and environment) origins, serotypes, FimH type, VFs, ARGs, and MGEs, and the structure of a novel ARG-carrying plasmid within the One Health context.

## RESULTS

### Complete genome sequencing of *E. coli* ST405 strains isolated in Pakistan

A total of three *E. coli* strains were isolated from sewage and animal feces across various regions of Pakistan (Table S1). These strains were classified as ST405 based on MLST analyses. The sequencing results and assembly statistics for these strains are summarized in Table S2. Two of these ST405 strains were isolated from sewage, while one was recovered from an animal host, highlighting the presence of this high-risk clone in both animal and environmental sectors in a low-income country (Table S1). All strains harbored the *bla*_NDM-5_ gene on their plasmids, pPEC1013-1, pPEC1020-1, and pPEC1021-3, corresponding to strains PEC1013, PEC1020, and PEC1021, respectively. These plasmids also carried various other ARGs, whereas pPEC1013-2, pPEC1013-3, pPEC1013-4, and pPEC1021-1 carried no ARGs, and pPEC1020-2 and pPEC1021-2 possessed only the *bla*_CMY-42_ gene.

### Obtaining ST405 genomes from the public database for comparative genomic analyses

To gain a comprehensive understanding of the genomic landscape of the high-risk clone ST405, including its phylogeny, sequence variations, and its genetic repertoire of ARGs, VFs, and MGEs, genomes belonging to ST405 were retrieved from the NCBI GenBank genome database (as of 10 June 2024). Among the 322,160 *E. coli* genomes available in the database, 257,515 non-redundant genomes were subjected to MLST analysis, successfully identifying STs for 253,404 genomes (98.4%). The most frequently genome-sequenced ST was ST11 (11.6%). The database included 24 high-risk clones of ExPEC (ST58, ST101, ST88, ST23, ST44, ST48, ST744, ST10, ST617, ST167, ST410, ST457, ST648, ST117, ST393, ST38, ST69, ST405, ST1193, ST131, ST12, ST73, ST95, and ST127) (11). Among these, ST131 (6.6%) and ST10 (4.2%) were the most dominant high-risk clones. ST405 ranked as the 21st most common ST overall and 13th most prevalent among high-risk clones of ExPEC (Table S3). According to a previous study, ST405 was identified as the third most common carrier of the *bla*_NDM-5_ gene, following ST410 and ST167 (8, 12) (Table S3). These findings underscore the clinical importance of ST405, but its complete genomic landscape and evolutionary dynamics remain insufficiently characterized. A total of 1,983 genomes were classified as ST405. To ensure data quality, genomes containing more than 300 contigs were excluded, resulting in a final set of 1,775 ST405 genomes for comparative genomic analyses. These genomes were analyzed together with the genomes of three ST405 isolates (strains PEC1013, PEC1020, and PEC1021) sequenced in this study (Table S4). For the analysis of host-source distribution, we focused on a subset of strains with available One Health isolation source information. This subset included 1,642 strains. Among these, 1,538 isolates were from human samples, whereas only 68 and 36 isolates originated from animal and environmental sources, respectively.

### Genomic landscape of *E. coli* ST405: phylogenome, origin, serotype, ARGs, and MGEs

**Fig 1:**
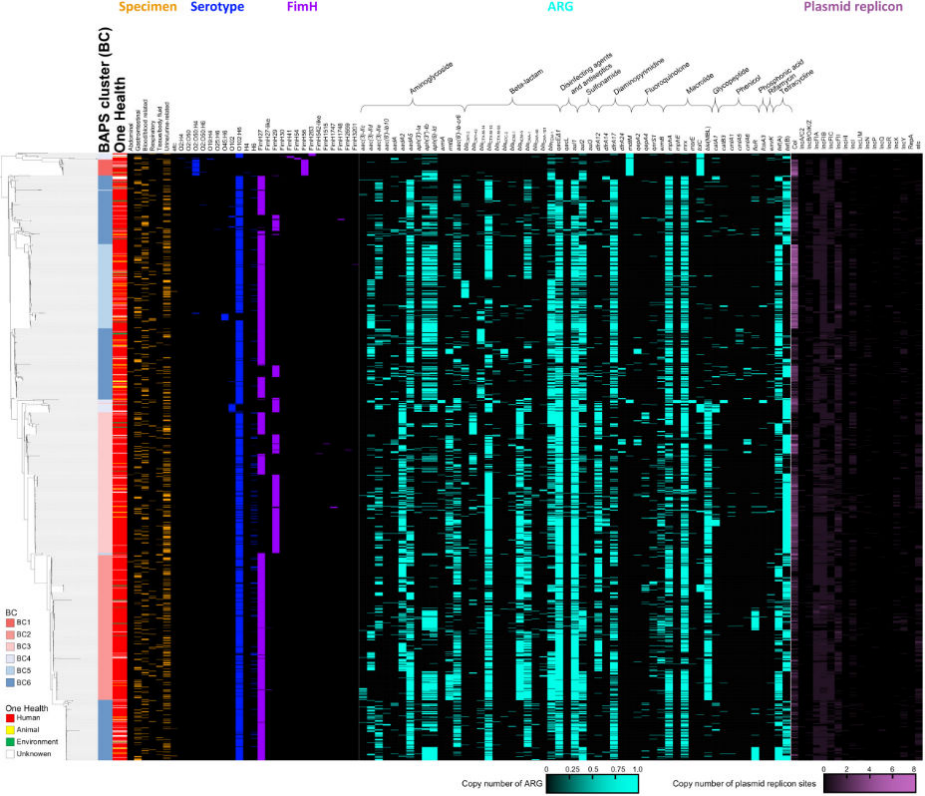
Genomic landscape of 1,778 *E. coli* ST405 genomes. The phylogenomic tree was constructed based on 2,548 core genes. BAPS clusters were determined using core-gene SNP-based distance calculation. One Health sector origin, specimen type, serotype, FimH type, the presence of ARGs, and the number of plasmid replicon sites are displayed as a heatmap.

A comprehensive phylogenomic analysis was conducted on *E. coli* ST405 using 1,778 genomes, which included 1,775 publicly available ST405 genomes retrieved from the database and three isolates sequenced in this study. The analysis was based on 2,548 core genes. *E. coli* strain 6722517 (NCBI RefSeq assembly accession no. GCF_033345115.1), belonging to ST69, was used as an outgroup (Fig. 1). To investigate the population structure of ST405, BAPS clustering was performed using core gene single-nucleotide polymorphism (SNP)-based distance calculation, which identified six distinct BAPS clusters (BCs) within the ST405 genomes. The clustering results were broadly congruent with the major lineages in the phylogenomic tree, though some discrepancies were observed. Such discrepancies likely reflect methodological differences: phylogenetic trees primarily model vertical inheritance, whereas BAPS clustering infers population structure from patterns of shared genetic variation that can be influenced by recombination and other non-vertical processes (Fig. 1). For instance, certain genomes in BC4 and BC6 formed a separate branch on the tree (Fig. 1). Among the isolates from this study, PEC1013 was classified into BC2, while PEC1020 and PEC1021 were assigned to BC3 (Table S4).

To further understand the distribution of ST405, metadata of genomes retrieved from the NCBI BioSample database, and the One Health sector origin and isolation specimen (for human isolates only) were incorporated into the phylogenomic tree. While 86.5% (1,538/1,778) of the analyzed ST405 genomes were derived from human sources, this likely reflects a bias in sequencing and reporting rather than the true ecological distribution of the lineage. Nonetheless, the detection of ST405 in animal and environmental reservoirs highlights its potential for cross-sectoral transmission within a One Health context. To evaluate the potential impact of clonality among the 1,778 ST405 genomes analyzed, we examined the metadata associated with year, country, and specimen type. Although 86.5% of the genomes originated from human clinical sources, only a small number of isolate groups shared identical metadata (isolation year, country, and human specimen origin) (Table S4). The most represented group consisted of 49 genomes collected from urine samples in the United States in 2023, followed by smaller groups with 31 (United States, 2019), 24 (Japan, 2019), and 22 (United States, 2022) genomes, respectively (Fig. S1). To determine whether these metadata-overlapping isolates represent clonal expansions, we projected them onto the core-genome phylogenetic tree. While limited local clustering was observed, the majority of these isolates were phylogenetically dispersed across multiple branches, indicating substantial genomic diversity even within metadata-identical subsets (Fig. S1). These findings suggest that the overall influence of clonality on comparative analyses is minimal.

When considering specimen types among human isolates, ST405 was frequently detected in urine (23.6%), followed by blood (12.6%), and GI tract (12.6%), supporting its classification as a major ExPEC lineage associated with urinary tract infections (15). However, no significant association was found between isolation sources (including specimen types among human isolates) and phylogenomic clustering (Fig. 1).

Serotype prediction analysis revealed 11 distinct serotypes among ST405 genomes, with O102:H6 being the most predominant. This serotype was detected across all BCs except for BC1, where O2:O50:H4 was more prevalent (Fig. 1). The predominance of O102:H6 has been previously reported in ExPEC ST405 strains (16). FimH typing identified 14 distinct alleles within ST405 genomes, with FimH27 (72.3%) and FimH29 (22.6%) being the most frequently observed. Consistent with the serotype distribution, BC1 was associated with FimH56, while BC3 displayed a distinct FimH29-dominant profile (67.5%). In contrast, FimH27 was predominant in BC2, BC4, BC5, and BC6 (Fig. 1; Table S4).

Further investigation into the genetic composition of ST405 focused on VFs and ARGs, and their presence was incorporated into the tree. VF genes were grouped based on their functional classification. Most VF gene clusters were found to be highly conserved across ST405 strains. To highlight the pathogenic potential of ST405, we compared its genomic features with those of ST2178, a minor lineage including two wild-bird fecal isolates from Pakistan in a previous comparative genomic survey (17). This comparison revealed that ST405 strains exhibited a broader repertoire of VFs. No clear correlation was observed between phylogeny and VF repertoires within ST405 genomes (Fig. S2).

Analysis of ARGs identified a total of 250 ARGs across ST405 genomes using the Resistance Gene Identifier pipeline. Sublineage-variable ARGs were defined as those present in more than 1% of genomes but less than 90% of genomes in at least one BC, provided that the gene did not exceed 90% prevalence in any BC. ARGs with ≥90% prevalence in any BC were classified as sublineage-specific intrinsic for that BC and excluded from the sublineage-variable set, while ARGs detected in fewer than 1% of genomes across all BCs were regarded as negligible. Based on these criteria, 55 sublineage-variable ARGs were elucidated in ST405 genomes (Table S5), and their distribution was visualized in a heatmap (Fig. 1). Among these, 23 sublineage-variable ARGs were present in more than 10% of genomes and included aminoglycoside resistance genes [*aac(3)-IId*, *aac(3)-IIe*, *aadA2*, *aadA5*, *aph(3'')-Ib*, *aph(6)-Id*, *rmtB*, and *aac(6')-Ib-cr6*], β-lactam resistance genes (*bla*_CTX-M-15_, *bla*_NDM-5_, *bla*_OXA-1_, and *bla*_TEM-1_), a disinfecting agent resistance gene (*qacE*Δ*1*), sulfonamide resistance genes (*sul1* and *sul2*), diaminopyrimidine resistance genes (*dfrA12* and *dfrA17*), macrolide resistance genes (*ermB*, *mphA*, and *mrx*), a glycopeptide resistance gene [*ble*(MBL)], and tetracycline resistance genes [*tet(A*) and *tet(B*)] (Table S5, blue-colored ARGs). Among those, an ESBL gene (*bla*_CTX-M-15_), carbapenemase (*bla*_NDM-5_), and conserved ARGs associated with class 1 integron (*qacE*Δ*1* and *sul1*) were of particular concern. The frequent occurrence of both genes [*bla*_NDM-5_ and *ble*(MBL)] was found in ST405 genomes (Fig. 1), and this genetic linkage was confirmed in strains PEC1013, PEC1020, and PEC1021, where these genes were found on the same plasmid. Additionally, an IS*26*-flanked transposon together with class 1 integron carrying multiple ARGs (*sul2*, *sul1*, *mrx*, *mphA*, *bla*_TEM-1_, *qacE*Δ*1*, *aadA5*, and *dfrA17*) has been previously identified in ST405 (15). In this study, *sul1*, *mrx*, *mphA*, *bla*_TEM-1_, *qacE*Δ*1*, *aadA5*, and *dfrA17* were also detected in more than 40% of ST405 genomes, suggesting the likelihood that a Tn*6242*-like structure contributes to ARG dissemination within ST405 (Fig. 1; Table S5). Unlike VFs, ARG profiles in ST405 were found to be strongly associated with phylogenetic structure. For instance, BC1 exhibited a notably lower copy number of ARGs compared to the other clusters. Genes encoding BRP(MBL) and NDM-5 β-lactamase, which are frequently co-located, also displayed distinct phylogenetic clustering patterns and BC-specific distributions (Fig. 1).

**Fig 2:**
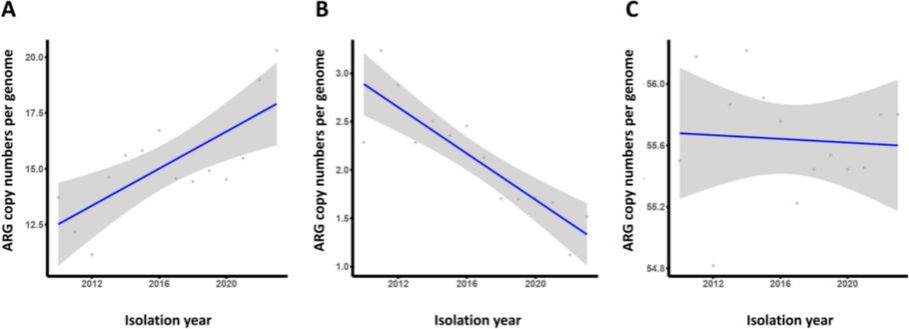
Time course increase analysis of ARG prevalence. The proportion of ARGs per year (2010–2023) was calculated based on the number of ARGs per strain isolated in each year. (**A**) Sublineage-variable ARGs with an occurrence rate exceeding 10% in genomes (slope = 0.415, *R*² = 0.538, *P*-value < 0.05); (**B**) sublineage-variable ARGs with an occurrence rate below 10% in genomes (slope = −0.12, *R*² = 0.763, *P*-value < 0.05); (**C**) non-sublineage-variable ARGs (slope = −0.006, *R*² = 0.005, *P*-value = 0.817).

A time-course analysis of the ARG copy number profile was conducted using linear regression, assessing the proportion of summation of ARGs per year (number of ARG copies per number of genomes isolated each year). ARGs were categorized into three groups: sublineage-variable ARGs exhibiting more than 10% occurrence (23 ARGs); sublineage-variable ARGs exhibiting less than 10% occurrence (32 ARGs); and non-sublineage-variable ARGs (195 ARGs) (Table S5). Among these groups, the first group (highly prevalent sublineage-variable ARGs) exhibited a strong positive correlation with time, with statistical significance (Fig. 2). The results suggested that these 23 sublineage-variable ARGs are actively disseminating and proliferating within the ST405 lineage, contributing to its increasing resistance profile over time.

Plasmid replicon sites were categorized into 18 groups. Among these, Col and IncFIB plasmids were the most frequently detected, with Col plasmids present at 2.06 (0–16) copies per genome. Notably, IncFIB plasmids have been previously identified as key carriers of the *bla*_NDM-5_ gene (18). The distribution of plasmid replicon sites was highly conserved across all ST405 genomes, suggesting widespread plasmid-mediated ARG transmission within this ST. In addition, MGEs were identified at an average of 31 elements per genome. The most frequently identified MGE was MITEEc1, which has been reported to be associated with VFs in *E. coli* (19). However, in contrast to ARGs, MGE profiles did not follow phylogenetic clustering (data not shown).

### Characteristics of BCs of ST405

Phylogenomic analysis revealed subtle clustering patterns of ARGs, MGEs, and VFs across BCs. To validate the statistical significance of these patterns, principal coordinate analyses (PCoA) were performed, followed by pairwise PERMANOVA testing (Fig. S3 to S5), all of which yielded significant differences between BAPS clusters (*P*-value <0.001). In the PCoA of VFs, BC5 exhibited the greatest divergence compared to BC2, with pseudo-*F* values of 793.9 (Fig. S3C). For plasmid replicons, the most significant distinction was observed between BC1 and BC2 (pseudo-*F* value of 27.3) (Fig. S4C). Regarding sublineage-variable ARGs, BC1 showed clear differentiation from BC2 (pseudo-*F* value of 243.3), and also BC3 was strongly distinct from BC1 (pseudo-*F* value of 242.7) (Fig. S5C). BC1 exhibited a higher copy number of VFs but a lower copy number of ARGs (Fig. S3A and S5A). BC1 was characterized by an enrichment of VFs including P fimbriae, the secreted autotransporter toxin (Sat) cluster, α-hemolysin, *Shigella* enterotoxin 2, and TraJ (Fig. S3D).

In contrast, sublineage-variable ARGs were distinct across BCs, revealing a clear separation between BC2, BC3, and BC4 versus BC5 and BC6. While the overall abundance of ARGs was comparable among BC2, BC3, BC4, and BC6, notable differences in composition were observed (Fig. 1; Fig. S5D). Specific aminoglycoside resistance genes such as *aadA5*, *aph(3'')-Ib*, and *aph(6)-Id*, and a diaminopyrimidine resistance gene *dfrA17* were found at higher frequencies in BC5 and BC6. Conversely, *bla*_NDM-5_, *ble*(MBL), *aadA2*, and *dfrA12* genes showed high prevalence exclusively in BC2, BC3, and BC4 (Fig. 1; Fig. S5D). Based on differences in ARG repertoire and composition, BC1 was categorized as subgroup A, BC2, BC3, and BC4 were grouped as subgroup B, and BC5 and BC6 were classified as subgroup C (Table S4).

### Characteristics of ST405 subgroups

**Fig 3:**
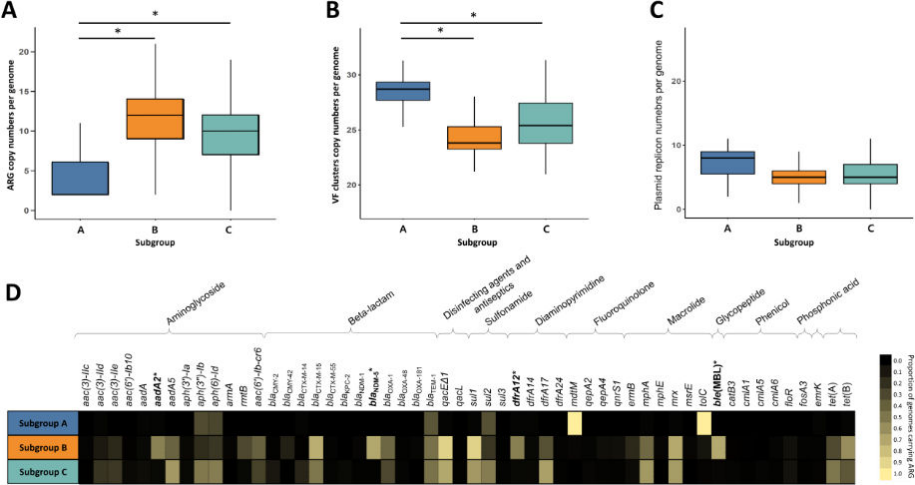
Sublineage-variable ARGs, VF gene clusters, and plasmid replicon repertoires across subgroups. (**A**) Copy number of sublineage-variable ARG occurrences per genome; (**B**) copy number of VF gene clusters per genome; (**C**) copy number of plasmid replicon sites per genome; (**D**) heatmap representation of sublineage-variable ARG prevalence (proportion of genomes carrying each gene) across subgroups. For panels **A–C**, subgroup differences were assessed using the Kruskal-Wallis test, and significant pairwise comparisons are indicated by asterisks (*P*-value < 0.001). For panel **D**, differences in gene prevalence across subgroups were assessed using the χ^2^ test, and significant genes are marked by asterisks (*P*-value < 0.001).

**Fig 4:**
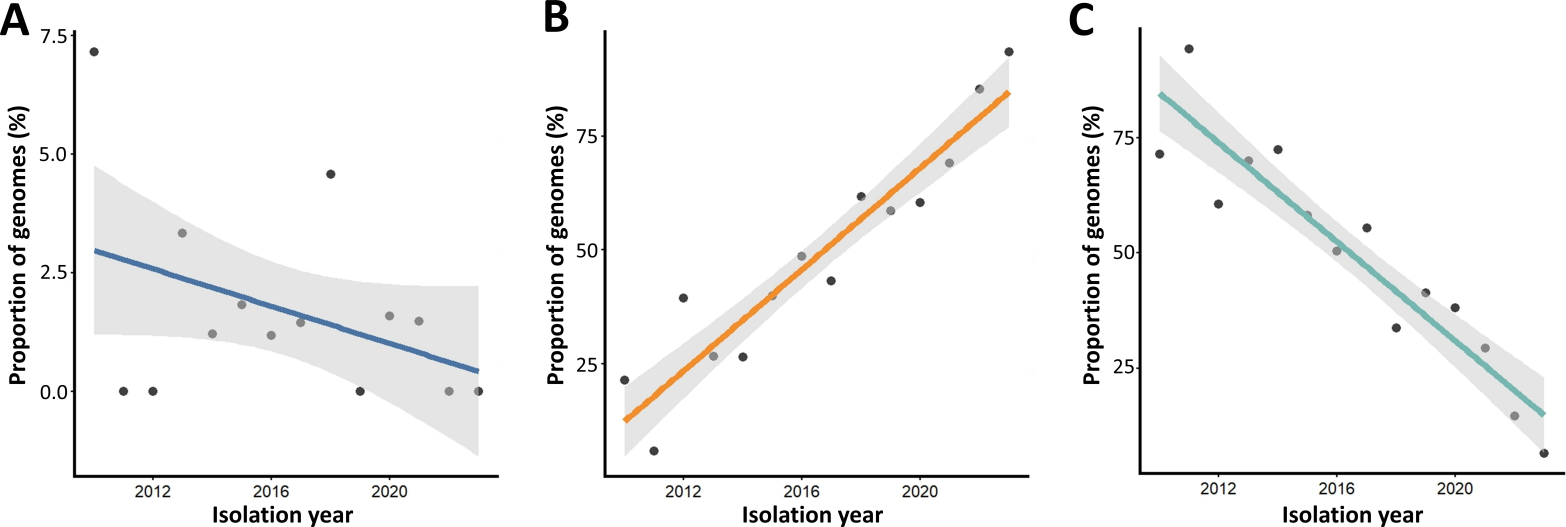
Temporal distribution of genome-sequenced ST405 isolates across subgroups. (**A**) Subgroup A; (**B**) subgroup B; (**C**) subgroup C. For each year from 2010 to 2023, the *y*-axis shows the annual proportion (%) of genomes assigned to each subgroup among all ST405 genomes available for that year. Linear regression was performed to evaluate temporal trends, and the significance of the regression slope was assessed using a *t*-test (subgroup A: slope = −0.20, *R*² = 0.158, *P*-value = 0.16; subgroup B: slope = 7.22, *R*² = 0.523, *P*-value = 0.003; subgroup C: slope = −5.37, *R*² = 0.869, *P*-value < 0.001).

To further characterize the ST405, genome-level copy numbers of ARGs, VFs, and plasmid replicons were compared across subgroup A (BC1), subgroup B (BC2, BC3, and BC4), and subgroup C (BC5 and BC6). Subgroup A exhibited a distinct genomic profile, characterized by a higher copy number of VFs and a lower copy number of ARGs compared to subgroups B and C. These subgroup-level differences were statistically supported (Kruskal-Wallis test; Fig. 3A and B). In contrast, subgroups B and C displayed similar overall amounts of ARGs, VFs, and plasmid replicons (Fig. 3A through C). However, they were distinguished by their ARG composition, with subgroup B exhibiting a higher prevalence of *aadA2*, *bla*_NDM-5_, *dfrA12*, and *ble*(MBL) genes, which was statistically supported (χ^2^ test; Fig. 3D). Strains PEC1013, PEC1020, and PEC1021 were classified into subgroup B, as they harbored the *bla*_NDM-5_ gene in their plasmids, all of which belong to the IncF incompatibility group, reinforcing their classification as carbapenemase-producing ST405 isolates. Across all subgroups, human-derived isolates were predominant. Although the proportions of urine- and blood-derived specimens varied slightly between subgroups, there was no meaningful difference in specimen type distribution. There was no clear distinction between subgroups and isolation origin. To assess the epidemiological trajectory of subgroup B, which exclusively harbors the *bla*_NDM-5_ gene, the temporal emergence of strains belonging to each subgroup was analyzed in a time course. The population representation of subgroup B among ST405 strains was analyzed over time using linear regression modeling. To minimize potential sampling bias arising from temporal variation in sequencing volume, subgroup prevalence was calculated as the annual proportion of genomes assigned to each subgroup among all ST405 genomes available for that year. The results demonstrated a statistically significant increase in the prevalence of subgroup B over time, whereas subgroup A showed no significant temporal trend, and subgroup C showed a significant decrease over time (Fig. 4). These findings indicate that subgroup B, characterized by the exclusive presence of *bla*_NDM-5_, is emerging as the dominant ARG-associated genotype within ST405, reinforcing concerns regarding its increasing role in global antimicrobial resistance dissemination.

**Fig 5:**
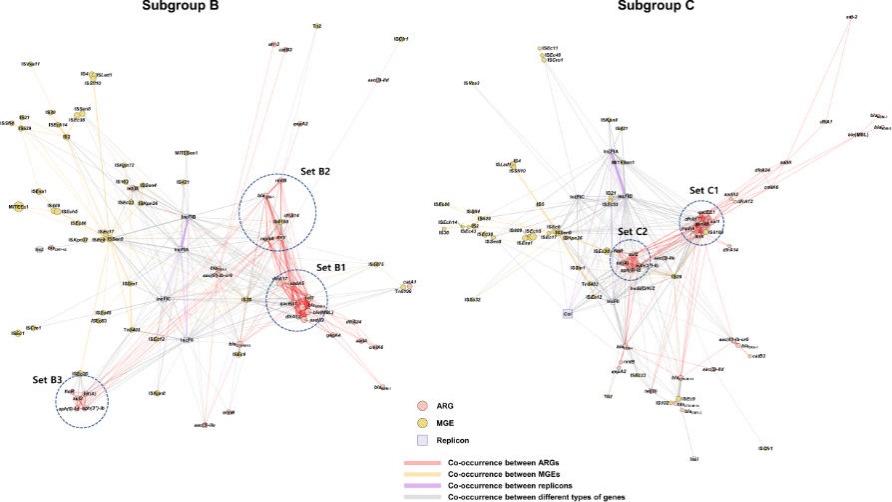
Co-occurrence networks of ARGs, MGEs, and plasmid replicon types in ST405 subgroups. Co-occurrence networks were constructed for subgroup B (left) and subgroup C (right). Nodes represent ARGs (pink circles), MGEs (yellow circles), and plasmid replicon types (purple squares). Edges indicate pairs of genetic elements (ARG-ARG, ARG-MGE, and MGE-MGE) that co-occur within ±10 kb on the same contig, whereas ARG-replicon and MGE-replicon edges indicate ARGs or MGEs located on contigs carrying the corresponding plasmid replicon. Only pairs with a co-occurrence frequency of ≥1% within each subgroup are shown, and edge width is proportional to this frequency. Node positions were optimized using the ForceAtlas2 layout algorithm in Gephi. Dashed circles highlight subgroup-specific core sets.

To understand how these subgroup-specific ARG profiles are organized on MGEs and plasmids, we performed subgroup-level co-occurrence network analysis based on 47, 896, and 835 genomes from subgroups A, B, and C, respectively (Fig. 5; Fig. S6). In these networks, each node represents an ARG, MGE, or plasmid replicon, and edges represent pairwise co-occurrence among these elements. In subgroup A, where both the number of genomes and the overall abundance of acquired ARGs were low, the co-occurrence network was sparse, and only a few ARGs [*sul2*, *bla*_TEM-1_, *aph(6)-Id*, and *aph(3'')-Ib*] showed weak links to MGEs or replicons, without a clearly defined core module (Fig. S6). In contrast, both subgroups B and C exhibited dense ARG-MGE-replicon networks, indicating that complex resistance modules are present in ST405 sublineages (Fig. 5). However, the composition of the central network core differed between the two subgroups. In subgroup B, the innermost core (set B1) was formed by *sul1*, *qacE*Δ*1*, *aadA5*, *dfrA17*, *dfrA12*, *aadA2*, *ble*(MBL), and *bla*_NDM-5_. A second layer (set B2) of closely connected nodes was composed of *rmtB*, *mrx*, *mphA*, *dfrA14*, and *bla*_TEM-1_, whereas a more peripheral module (set B3) contained *sul2*, *floR*, *tet(A*), *aph(6)-Id*, and *aph(3'')-Ib*. Sets B1 and B2 showed frequent co-occurrences with IS*26* and IncF-type plasmid replicons, indicating that these resistance determinants are embedded in *IS*26-rich IncF plasmid backbones. IS*6100* was most strongly connected to the set B2 genes, particularly *mphA*, *mrx*, and *bla*_TEM-1_. In subgroup C, the overall network density was comparable. The central core (set C1) consisted of *sul1*, *qacE*Δ*1*, *aadA5*, *dfrA17*, *mphA*, and *mrx*, which formed a highly connected ARG cluster showing strong co-occurrence with IS*6100*. A second, more peripheral module (set C2) included *sul2*, *floR*, *tet(A*), *aph(6)-Id*, and *aph(3'')-Ib*. Both sets C1 and C2 were frequently linked to IS*26* and IncF-type replicons, again pointing to IncF plasmids as major carriers of these resistance modules. Notably, the *bla*_NDM-5_–*ble*(MBL) cluster, along with accompanying *aadA2* and *dfrA12*, which define the set B1 core in subgroup B, was absent from the subgroup C core. Taken together, subgroups B and C share a broadly similar ARG-MGE-plasmid framework centered on IS*26*- and IS*6100*-associated IncF plasmids. However, subgroup B is uniquely characterized by an additional *bla*_NDM-5_-containing core module, which may contribute to its emerging dominance among ST405 lineages.

### Subgroup B: major *bla*_NDM-5_ carrier group

**Fig 6:**
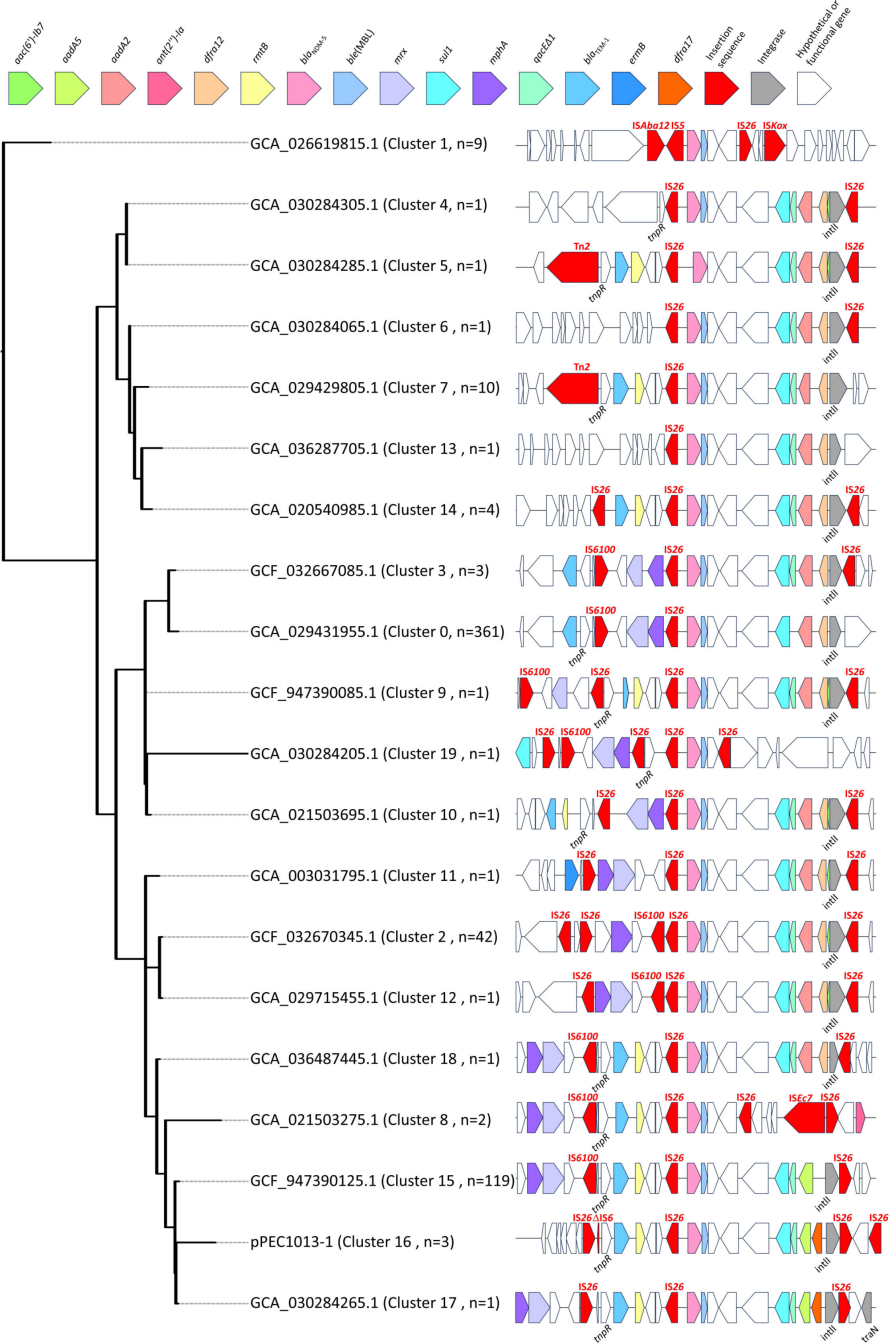
Phylogenetic tree of 20 representative *bla*_NDM-5_ gene clusters, including intact 10 kb upstream and downstream sequences and their genetic structures. The phylogenetic tree was constructed using the maximum-likelihood algorithm. Cluster 1, carrying the fewest ARGs, was used as an outgroup. NCBI genome assembly accession numbers of representative sequences are provided in the tree.

To investigate the genetic context of the *bla*_NDM-5_ gene in subgroup B, genomes containing intact 10 kb upstream and 10 kb downstream flanking regions (total 20 kb) of the gene were selected, resulting in 29 genomes, including strains PEC1013, PEC1020, and PEC1021. These sequences from 29 genomes were clustered, yielding 20 representative sequences of *bla*_NDM-5_ genetic context. A maximum-likelihood phylogenetic tree was constructed using these 20 representative sequences (Fig. 6). Cluster 1 was designated as an outgroup, as it harbored the fewest ARGs. All analyzed sequences contained a class 1 integron structure adjacent to the *bla*_NDM-5_ gene, with frequent insertion of various insertion sequences (ISs), including IS*26*. Comparative analysis of the *bla*_NDM-5_ genetic contexts revealed that the gene was consistently flanked by IS*26* and integrated at a position beyond the 3′-conserved segment of a class 1 integron, suggesting that IS*26* mediates the mobilization of *bla*_NDM-5_ within ST405 (Fig. 6). To assess the prevalence of these genetic structures, the analysis was expanded to include a total of 564 genomes containing partial *bla*_NDM-5_ flanking regions (at least 5 kb both upstream and downstream). Among the identified structures, cluster 0 was the most prevalent, detected in 361 genomes, followed by cluster 15 (119 genomes) and cluster 2 (42 genomes). These dominant clusters accounted for the majority of the analyzed data set, while other clusters were observed at lower frequencies. The plasmids of strains PEC1020 (pPEC1020-1) and PEC1021 (pPEC1021-3) carrying the *bla*_NDM-5_ gene were classified as cluster 0. The tree revealed distinct clustering patterns based on ARG composition. Clusters 4, 5, 6, 7, 13, and 14 lacked *mrx* and *mphA* genes, which are associated with macrolide resistance. Clusters 15, 16, and 17 uniquely carried *aadA5* genes, whereas other clusters predominantly harbored *aadA2* (Fig. 6). The plasmid of strain PEC1013 (pPEC1013-1), which carried the *bla*_NDM-5_ gene, formed a unique genetic cluster (cluster 16), exhibiting a distinct genetic structure (Fig. 6).

Plasmids pEC1020-1 and pPEC1021-3 were identical except for a single base pair insertion observed in pPEC1020-1 (Table S6). Highly similar plasmids, identified by whole-plasmid comparisons exhibiting more than 95% query coverage and 99% sequence identity to pPEC1020-1 and pPEC1021-3, were frequently detected in *E. coli* ST405 strains (9 out of 19 strains), ST156 (6 of 19 strains), and ST648 (2 of 19 strains). The plasmid displayed a global distribution irrespective of geographical locations, with most strains isolated from urine samples. This suggests a significant prevalence of these plasmids within these ExPEC STs and highlights their role as carriers of the *bla*_NDM-5_ gene in specific STs (Table S6).

**Fig 7:**
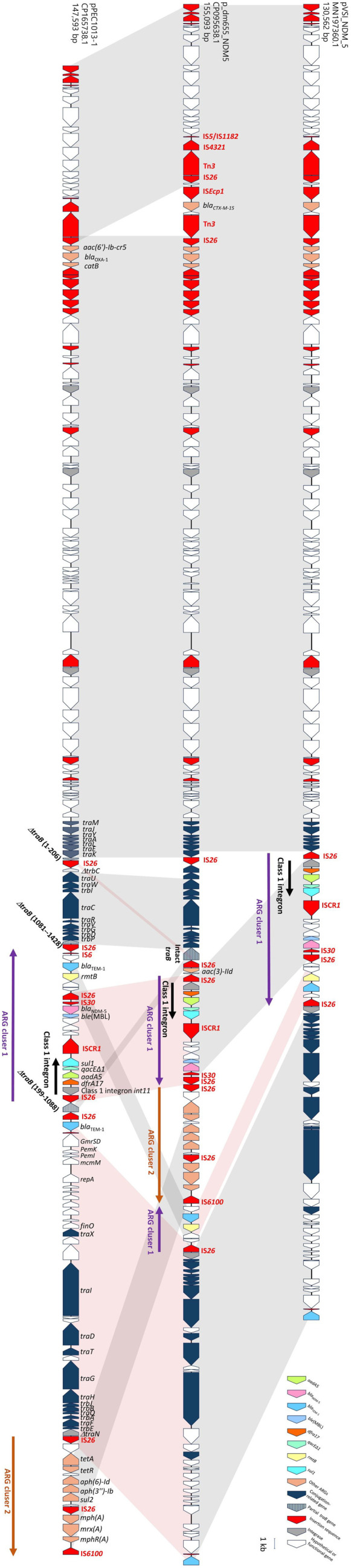
Comparative genetic structures of plasmids pPEC1013-1, p_dm655_NDM5, and pVSI_NDM_5. Shared regions with >99% identity are shaded. Gray and pink shadings represent the orientation of shared regions, indicating directed and inverted alignments, respectively.

**Fig 8:**
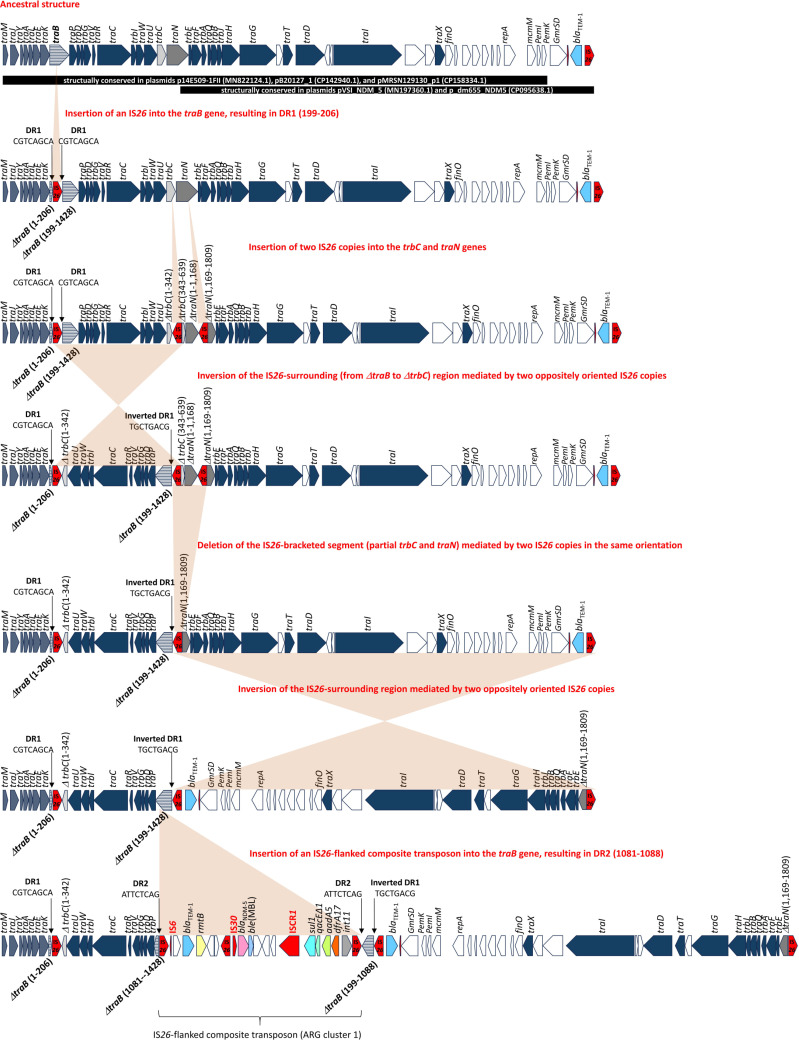
Proposed IS*26*‐mediated rearrangement of the *tra* region in plasmid pPEC1013-1. From top to bottom, the panels illustrate a parsimonious sequence of IS*26*-mediated events starting from an inferred ancestral F plasmid-like *tra* backbone. In the ancestral structure, the segment from *traM* to *pemK* is conserved in several IncF plasmids, and the segment from Δ*traN* to *bla*_TEM-1_ is conserved in other *bla*_NDM-5_-carrying plasmids; their continuous collinearity was used to reconstruct the ancestral tra region (top panel). An IS*26* insertion into *traB* generates the first 8-bp target-site duplication DR1 (positions 199–206) (second panel). Two additional IS*26* copies insert independently into *trbC* and *traN* (third panel). Recombination between the oppositely oriented IS*26* copies in *traB* and *trbC* inverts the Δ*traB–*Δ*trbC* segment, producing an inverted copy of DR1 (fourth panel). Recombination between IS*26* copies in the same orientation deletes the IS*26*-bracketed region, removing parts of *trbC* and *traN* (fifth panel). A further inversion between the remaining oppositely oriented IS*26* copies inverts the segment spanning Δ*traN* to *bla*_TEM-1_, reversing the orientation of this block relative to the conserved *tra* backbone (sixth panel). Finally, an IS*26*-flanked composite transposon carrying ARG cluster 1 inserts into Δ*traB*, generating the second 8-bp target-site duplication DR2 (positions 1081–1088) (bottom panel). Genes are shown as arrows, IS*26* copies are highlighted in red, and shaded areas indicate segments that are inverted or deleted between successive steps.

On the contrary, an identical sequence of plasmid pPEC1013-1 was not found in the NCBI GenBank database, consistent with the results as shown in Fig. 6 (cluster 16). Only two similar plasmids, pVSI_NDM_5 and p_dm655_NDM5, were identified. Plasmid p_dm655_NDM5 (GenBank accession number, NZ_CP095638.1) exhibited 100% query coverage and 99.99% identity with pPEC1013-1, while plasmid pVSI_NDM_5 (NZ_MN197360.1) showed 86% query coverage and 99.98% identity with pPEC1013-1. Plasmid p_dm655_NDM5 was identified in a ST405 strain (*E. coli* strain dm655, JAGIHN000000000.1), but the ST information for the strain carrying plasmid pVSI_NDM_5 was not available. Both plasmids were classified as IncF-type plasmids. They originated from human samples, whereas plasmid pPEC1013-1 was isolated from animal feces. Comparative analyses revealed the primary variations among the three plasmids were located within the conjugation factor (*tra*) gene clusters. Although the class 1 integron structure harboring *dfrA17*, *aadA5*, *qacE*Δ*1*, and *sul1* genes was conserved among the plasmids, their genetic contexts within the *tra* gene cluster differed. In plasmid pVSI_NDM_5, ARG cluster 1, IS*26*-flanked region carrying the class 1 integron, *ble*(MBL), *bla*_NDM-5_, *rmtB*, and *bla*_TEM-1_, was located between *traE* and a truncated *traN* gene (Fig. 7). In plasmid p_dm655_NDM5, ARG cluster 2 was inserted into ARG cluster 1. The inserted module contained multiple resistance genes [*tet(A*), *sul2*, *mphA*, and *mrx*] and was flanked by IS*26* and IS*6100*. The insertion was accompanied by an inversion involving IS*6100* and an IS*26*-flanked region carrying *bla*_TEM-1_ and *rmtB*. This combined cluster was located between intact *traB* and truncated *traN* genes, with an additional insertion of *aac(3)-IId* upstream of ARG cluster 1 (Fig. 7). In plasmid pPEC1013-1, ARG cluster 1 was mostly conserved, except for variation in the region of *bla*_TEM-1_ and *rmtB* genes, whereas ARG cluster 2 was located separately from ARG cluster 1. Unlike other plasmids, plasmid pPEC1013-1 exhibited a distinct insertion of ARG cluster 1 within the *tra* gene (Fig. 7). The distinct genetic structure of plasmid pPEC1013-1 was scrutinized to reveal the genetic variation surrounding the *bla*_NDM-5_ gene context in the ST405 strain. In plasmid pPEC1013-1, the architecture of the *tra* region and ARG cluster 1 can be explained by a series of IS*26*-mediated rearrangements (Fig. 8). To define the starting point for this model, we first reconstructed a hypothetical ancestral *tra* region by aligning pPEC1013-1 with related plasmids. The segment from *traM* to *pemK* is continuously conserved in several plasmids including p14E509-1FII (MN822124.1), pB20127_1 (CP142940.1), and pMRSN129130_p1 (CP158334.1), whereas the segment from Δ*traN* to *bla*_TEM-1_ is structurally conserved in plasmids pVSI_NDM-5 (MN197360.1) and p_dm65_NDM5 (CP095638.1) analyzed in Fig. 7. Because these two conserved segments can be placed in direct succession and a similar joined configuration is observed in related IncF plasmids, we considered their concatenation to be a parsimonious approximation of the ancestral F plasmid-like *tra* backbone carrying ARG cluster 1, which served as the starting point for our reconstruction (top panel of Fig. 8). Starting from this ancestral configuration, the first event was the insertion of an IS*26* element into *traB*, generating an 8-bp target-site duplication (DR1; positions 199–206) within the gene (second panel of Fig. 8). An 8-bp target-site duplication is the characteristic signature of *IS*26 copy-in transposition (20, 21). Two additional IS*26* copies then independently inserted into *trbC* and *traN*, creating a configuration in which IS*26* elements flanked the Δ*traB–*Δ*trbC* interval in opposite orientations (third panel of Fig. 8). Similar arrays of multiple IS*26* copies, including those with mixed orientations within conjugation and resistance regions, have been reported in multidrug-resistant plasmids (20, 22). Recombination between these oppositely oriented IS*26* copies would invert the Δ*traB–*Δ*trbC* segment, resulting in the observed inverted copy of DR1 (fourth panel of Fig. 8). Similar insertion sequence-mediated inversions of transfer or resistance modules have been reported, including an IS*1*-mediated inversion involving *traB* on plasmid pCMY42_EC8 (23) and IS*26*-associated inversions of multidrug-resistance regions in *Salmonella* chromosomal genomic islands (24). A subsequent recombination event between IS*26* elements in the same orientation would delete the internal IS*26*-bracketed region, removing parts of *trbC* and *traN* (fifth panel of Fig. 8). Comparable deletions of IS*26*-bounded segments, interpreted as movement or resolution of pseudo-compound transposons formed by directly oriented IS*26* copies and involving IS*26* transposition together with homologous recombination, have been reported (20, 25). A further inversion between the remaining oppositely oriented IS*26* copies would invert the IS*26*-bracketed segment spanning from Δ*traN* to *bla*_TEM-1_, thereby reversing the orientation of this block relative to the conserved *tra* backbone (sixth panel of Fig. 8). Similar IS*26*-associated inversions of adjacent or multidrug-resistance segments have been described in clinical plasmids and chromosomal resistance regions (22, 24, 26). Finally, insertion of an IS*26*-flanked composite transposon carrying ARG cluster 1 into Δ*traB* would produce the second 8-bp target-site duplication (DR2; positions 1081–1088). IS*26*-flanked composite transposons that introduce multidrug-resistance modules into plasmid backbones have been reported previously (27, 28). This model accounts for the presence and orientation of all IS*26* copies, the DR1/DR2 target-site duplications, and the characteristic truncations of *traB*, *trbC*, and *traN* in pPEC1013-1. These observations are compatible with the notion that IS*26*-driven replicative transposition can progressively remodel conjugative backbones and adjacent resistance regions in multidrug-resistant plasmids (22).

## DISCUSSION

According to the World Health Organization, ExPEC is one of the leading causes of urinary tract infections and bloodstream infections, with its prevalence steadily increasing globally (29). The pathogenicity of *E. coli* is driven by VFs, which vary among different pathotypes (2). While extensive research has been conducted to identify major VFs, pathotype-specific VFs remain unclear, often limiting evaluations to general pathogenic potential. This challenge necessitates comprehensive studies that integrate genomic structures and metadata to better characterize the pathogenic potential of *E. coli*. Alongside pathogenicity, antibiotic resistance poses a significant concern. Although *E. coli* is generally susceptible to antibiotics, it acquires ARGs through HGT, mediated by MGEs including plasmids (7, 18, 30). Clinical reports have identified cases of pathogenic *E. coli* harboring plasmids encoding multiple ARG copies in patients with prolonged infections (31). This highlights the bacterial ability to adapt under antibiotic selective pressure, underscoring the importance of investigating ARG dynamics alongside VFs.

To gain insights into the genomic characteristics of high-risk clones, comparative genomic analyses were conducted on *E. coli* strains belonging to the high-risk clone ST405. The temporal increase of major sublineage-variable ARGs in ST405 genomes emphasizes the importance of this ST in ARG dissemination. In our analysis, several sublineage-variable ARGs with low overall prevalence (<10%) exhibited a gradual decline over isolation years. This pattern contrasts with the stable or increasing frequency observed among more widespread resistance genes such as *bla*_CTX-M-15_ and *bla*_NDM-5_. While these rare ARGs spanned a range of antibiotic classes, they were inconsistently associated with BAPS clusters or replicon types. These findings suggest that their presence likely reflects sporadic acquisition events or transient introductions mediated by unstable plasmid backgrounds. Unlike high-prevalence ARGs that are often embedded in epidemic plasmids and subject to sustained selection, low-prevalence ARGs may be less competitively maintained within the ST405 population. Furthermore, ARG repertoires of ST405 include clinically significant genes such as *bla*_CTX-M-15_ and *bla*_NDM-5_, conferring resistance against critical antibiotics such as cephalosporins and carbapenems. In the case of the *bla*_CTX-M-15_ gene, it has been widely recognized as a key ESBL determinant (10) and is one of the most prevalent ARGs detected in *E. coli* isolates across the One Health sectors (32). Strains belonging to ST131, another high-risk clone, have disseminated globally as a major carrier of *bla*_CTX-M-15_ (33), and this gene has also been specifically associated with ST405 (10). In the present study, the *bla*_CTX-M-15_ gene was detected in more than 60.2% of ST405 genomes, whereas other *bla*_CTX-M_ variants exhibited lower frequencies (*bla*_CTX-M-14_, 8.0%; *bla*_CTX-M-55_, 2.0%) (Table S5). Similarly, the gene *bla*_NDM-5_ was frequently identified in ST405 genomes, consistent with findings from the European Centre for Disease Prevention and Control (8). A previous study reported that *bla*_NDM-1_ is frequently co-expressed with *ble*(MBL) under the same promoter (34). We note that the validity of our definition of sublineage-variable ARGs is dependent on the evolutionary and epidemiological resolution at which BCs are defined. In this study, BCs were delineated using fastBAPS clustering of 2,548 core genes, which provided stable sublineage structure across the data set. However, if BCs were defined at a much finer scale, even mobilizable ARGs could appear fixed within those clusters. Thus, the reproducibility of our classification framework depends on the granularity of the sublineage definition, and care should be taken when applying this approach to data sets with different evolutionary scales.

To further resolve the resistome-level structure beyond core genome phylogeny, we classified the ST405 genomes into three resistome groups (subgroups A–C) based on overall similarity in acquired ARG profiles. This additional layer of classification was motivated by the observation that certain BAPS clusters harbored highly similar resistome repertoires, despite their distinct positions in the core genome phylogeny. Because horizontal gene transfer can rapidly introduce shared ARGs across phylogenetically distinct backgrounds, accessory genome-based grouping enables functional clustering that is not evident from core genome structure alone. Importantly, the network analyses indicate that these resistome subgroups do not simply reflect the presence or absence of a single ARG, but instead correspond to distinct ARG-MGE-plasmid modules. In subgroup B, the enriched genes *bla*_NDM-5_, *ble*(MBL), *dfrA12*, and *aadA2* (Fig. 3D) frequently co-occurred with *sul1*, *qacE*Δ*1*, dfrA17, and aadA5, and this set was repeatedly associated with IS*26*- and IS*6100*-embedded IncF plasmid backbones (Fig. 5). Subgroup-level co-occurrence networks further show that this set and its co-located ARGs form the innermost core of the ARG-MGE-replicon network in subgroup B, tightly connected to IS*26*, IS*6100*, and IncF replicons, whereas the network core of subgroup C is instead centered on *sul1*, *qacE*Δ*1*, *dfrA17*, *aadA5*, *mphA*, and *mrx* and lacks the *bla*_NDM-5_–*ble*(MBL) module (Fig. 5). Notably, subgroup B also shows a distinct pattern of expansion over time, suggesting that this *bla*_NDM-5_-containing module confers an epidemiological advantage that facilitates the clonal and plasmid-mediated spread of ST405. Thus, resistome-based grouping, when integrated with information on MGEs and plasmid replicon types, provides a complementary and epidemiologically informative framework for characterizing ARG trends within clonal lineages such as ST405. Furthermore, the weak concordance between core genome-based BAPS clustering and the distribution of ARGs/MGEs highlights the dynamic nature of horizontal gene transfer. These events are often decoupled from the clonal phylogeny. Although homologous recombination may obscure phylogenetic signals, its influence is expected to be minimal within narrowly defined lineages such as specific sequence types, where clonal inheritance dominates the core genome structure (35).

Plasmids of the two environmental isolates of this study (pPEC1020-1 and pPEC1021-3), belonging to the most prevalent *bla*_NDM-5_ genetic context (cluster 0), were almost identical to plasmids found in ST156 and ST648 (a high-risk clone). These two STs have been reported as virulent and multidrug-resistant lineages, frequently isolated as uropathogenic strains (36, 37). The presence of the identical plasmids in ST405, ST156, and ST648 suggests a significant connection among these ExPEC STs in ARG transmission. Notably, the novel ARG cluster structure and unique insertion events by IS*26* replicative transposition were identified from an animal isolate. Numerous studies have reported the role of IS*26* in the dissemination of ARGs (20, 26). Understanding these IS*26*-mediated events provides crucial insights into ARG transmission mechanisms in *E. coli*. Remarkably, this novel structure was identified in a strain isolated from an animal, suggesting dynamic evolutionary changes in the *bla*_NDM-5_ gene context across isolates from One Health sectors. These findings emphasize the importance of surveillance of *E. coli* ST405 from the One Health perspective (14, 38, 39). The results contribute valuable insights into the epidemiological and clinical significance of this high-risk clone ST405, emphasizing the need for continued surveillance and research to address its impact on public health.

## MATERIALS AND METHODS

### Sampling and isolation of *E. coli* strains

Information about the isolation sources is provided in Table S1. To process sewage samples, samples were centrifuged to remove particles, and 100 µL of the supernatant was collected. The supernatant was then spread onto MacConkey agar plates supplemented with cefotaxime at a concentration of 1 μg/mL. To isolate feces-associated *E. coli* from animals, fresh fecal droppings of wild birds were collected from eight nesting and foraging sites in the Islamabad region, including urban parks, ecological settings, and hospital waste dumps. Samples were collected individually using sterile spatulas into autoclaved Eppendorf tubes pre-filled with phosphate-buffered saline, avoiding contact with soil or debris to minimize contamination. The tubes were sealed and transported on ice to the laboratory within 30–40 minutes. At the lab, 70 µL of each sample was serially diluted and plated onto MacConkey agar. The plates were incubated at 37°C for 18–24 hours. Pink colonies were selected and subjected to biochemical assays to confirm *E. coli* identity. Throughout the process, sterility was maintained by using sterile consumables and disinfecting instruments (17, 40, 41).

### Complete genome sequencing

Genomic DNA was extracted from the harvested *E. coli* cells from the culture using the MagAttract HMW DNA kit (Qiagen, Germany). To remove small fragments, AMPure XP bead purification was performed. For long-read sequencing, SMRTbell libraries were prepared using the SMRTbell Express kit (Pacific Biosciences, USA), and sequencing was conducted using the Sequel Sequencing Kit v.3.0 and SMRT Cell 1M v.2 on the PacBio Sequel sequencing platform (Pacific Biosciences, USA). The obtained sequencing data were assembled using the SMRT Link software (Pacific Biosciences, USA). For isolates that did not yield complete genome assemblies, additional sequencing was performed using the Illumina MiSeq platform (Illumina, USA). DNA fragments were processed using the TruSeq DNA Library LT kit (Illumina, USA) to construct MiSeq libraries, which were subsequently sequenced using the MiSeq Reagent Kit v.3 (Illumina, USA), generating 2 × 300 bp paired-end reads. A hybrid assembly of the MiSeq and PacBio sequencing data was performed using Unicycler version 0.4.9. To ensure the absence of barcode cross-contamination or misassignment in multiplexed sequencing runs, each assembly was screened using ContEst16S v.1.0, which detects unexpected 16S rRNA gene sequences (42). Genome annotation was performed using the Prokaryotic Genome Annotation Pipeline v.6.8 (43).

### Collection of ST405 genomes and their metadata from the NCBI databases

As of 10 June 2024, a total of 322,160 *E. coli* genome sequences were retrieved from the NCBI database using the NCBI data sets tool (v.16.20.0) with the selection criteria configured to include only annotated genomes (44). Corresponding metadata for each genome was obtained from the NCBI BioSample database using the Dataformat tool (v.16.20.0). Genomes with duplicate entries or those containing more than 300 contigs were excluded, and the remaining genomes were retained for subsequent analyses.

### Genome sequence-based analyses

STs of *E. coli* genomes were identified using the MLST tool (v.2.23.0, https://github.com/tseemann/mlst) based on the Achtman scheme. The analysis was conducted using seven housekeeping genes (*adk*, *fumC*, *gyrB*, *icd*, *mdh*, *purA*, and *recA*) (45).

For genome annotation in phylogenomic analyses, genomes, along with an outgroup genome (ST69 belonging to phylogroup D), were annotated using Prokka (v.1.14.6) (46). Roary (v.3.13.0) was run on Prokka-annotated GFF files to compute the ST405 pan-genome and generate the core-gene alignment (47). Roary identified 47,027 orthologous gene clusters: 2,548 core (≥99%), 846 soft core (95%–99%), 2,302 shell (15%–95%), and 41,331 cloud (<15%). The core-genome phylogeny was inferred from the concatenated alignment of the 2,548 core gene clusters. A total of 55,864 SNPs was identified from the alignment. This alignment was then used to construct a maximum-likelihood phylogenetic tree using FastTree (v.4.0.3) (48). The tree reflects sequence-based phylogenetic relationships and was visualized with iToL (v.7) (49).

To analyze population structure, the core-genome alignments were subjected to hierarchical Bayesian analysis of population structure using fastBAPS (v.1.0.8) (50). A conservative approach was applied by assuming a symmetric Dirichlet prior to prevent over-clustering in the phylogenomic tree, with only level 1 clustering results being considered.

ARGs were analyzed using RGI (v.6.0.3) with the “include nudge” option enabled. Only “perfect” and “strict” hits were retained for further analysis (51). VFs, serotypes, and plasmid replicon sites were annotated using ABRicate (v.1.0.0) (https://github.com/tseemann/abricate) with the VFDB (52), EcOH (53), and plasmidfinder (54) databases. FimH types were identified using FimTyper (v.1.0) (55), and MGEs, including IS elements and transposons, were analyzed using MEfinder (v.1.1.2) (56).

To assess the phylogenetic clustering of ST405 genomes, PCoA of ARG, VFs, and plasmid replicon sites for BCs was conducted using Bray-Curtis dissimilarity distances and visualized in R. The boundaries of each BC were visualized by calculating the covariance matrix of a normal distribution, with 95% confidence intervals used to define the regions. To examine temporal trends, linear regression analysis was performed using Student’s *t*-test and *R*² values to identify statistically significant relationships.

Structural variation surrounding the *bla*_NDM-5_ gene was characterized in two steps. First, to define representative genetic structures, genomes containing intact 10 kb upstream and 10 kb downstream flanking regions (total 20 kb) were selected. These sequences were clustered using CD-HIT (v.4.8.1) with a 95% identity threshold to identify structurally similar groups (57), and a dendrogram was generated with FastTree (v.4.0.3) (41). Second, to assess the prevalence of these structures, the analysis was expanded to include genomes harboring *bla*_NDM-5_ contigs with at least 5 kb flanking regions both upstream and downstream.

### Co-occurrence network analysis

Co-occurrence networks were constructed to explore the relationships among ARGs, MGEs, and plasmid replicon types within each ST405 subgroup. For each subgroup, nodes represented individual ARGs, MGEs, or plasmid replicon types. ARG-ARG, ARG-MGE, and MGE-MGE edges were defined when both elements were co-located within ±10 kb on the same contig, and ARG-replicon and MGE-replicon edges were defined when an ARG was present on a contig carrying the corresponding plasmid replicon. Co-occurrence frequency was calculated as the proportion of genomes in the subgroup in which each pair of nodes fulfilled these criteria, and only pairs with a frequency ≥1% were retained. Edge width was scaled according to this frequency. The ForceAtlas2 layout algorithm implemented in Gephi (v.0.10.1) was used to optimize node positions in the undirected networks (58, 59).

## Data Availability

*E. coli* genomes in this study are available at the NCBI BioSample accession numbers SAMN42567064, SAMN42418910, and SAMN42418969, for strains PEC1013, 1020, and 1021, respectively. The genome sequences of strain PEC1013 have been deposited in the NCBI GenBank database under accession numbers NZ_CP165737.1 (chromosome), NZ_CP165738.1 (plasmid pPEC1013-1), NZ_CP165739.1 (plasmid pPEC1013-2), NZ_CP165740.1 (plasmid pPEC1013-3), and NZ_CP165741.1 (plasmid pPEC1013-4). The genome sequences of strain PEC1020 have been deposited in the NCBI GenBank database under accession numbers NZ_CP162603.1 (chromosome), NZ_CP162604.1 (plasmid pPEC1020-1), and NZ_CP162605.1 (plasmid pPEC1020-2). The genome sequences of strain PEC1021 have been deposited in the NCBI GenBank database under accession numbers NZ_CP162406.1 (chromosome), NZ_CP162407.1 (plasmid pPEC1021-1), NZ_CP162408.1 (plasmid pPEC1021-2), and NZ_CP162409.1 (plasmid pPEC1021-3). The raw sequence data are available under Sequence Read Archive accession numbers SRX25649189 (MiSeq reads) and SRX25647294 (Sequel reads) for strain PEC1013, SRX25647293 (MiSeq reads) and SRX27926694 (Sequel reads) for strain PEC1020, and SRX27930644 (Sequel reads) for strain PEC1021.

## SUPPLEMENTAL MATERIAL

Fig. S1 msystems.01685-25-s0001.docx Genomic diversity of *E. coli* ST405 isolates of same isolation country, year, and human specimen origin.

Fig. S2 msystems.01685-25-s0002.docx Abundance profiles of virulence factors in *E. coli* ST405 genomes.

Fig. S3 msystems.01685-25-s0003.docx VF gene cluster repertoires in BAPS clusters.

Fig. S4 msystems.01685-25-s0004.docx Plasmid replicon sites repertoires in BAPS clusters.

Fig. S5 msystems.01685-25-s0005.docx Sublineage-variable ARG repertoires in BAPS clusters.

Fig. S6 msystems.01685-25-s0006.docx Co-occurrence networks of ARGs, MGEs, and plasmid replicon types in ST405 subgroup A.

Supplemental Tables msystems.01685-25-s0007.docx Tables S1 to S3, S5, and S6.

Table S4 msystems.01685-25-s0008.xlsx *E. coli* ST405 genomes used in this study and their information.

ASM does not own the copyrights to Supplemental Material that may be linked to, or accessed through, an article. The authors have granted ASM a non-exclusive, world-wide license to publish the Supplemental Material files. Please contact the corresponding author directly for reuse.
