# Supplementary material for: Comparative genomic analyses of Escherichia coli ST405 strains from Pakistan
Source: mSystems. 2026 Mar 16;11(4):e01685-25. doi: 10.1128/msystems.01685-25 (PMC13098264; doi:10.1128/msystems.01685-25)
Supplement: Fig. S1 — Genomic diversity of E. coli ST405 isolates of same isolation country, year, and human specimen origin. [file msystems.01685-25-s0001.docx]

**

**

**FIG S1** Genomic diversity of *E. coli* ST405 isolates of same isolation country, year and human specimen origin. Genomes with identical metadata were displayed in the phylogenomic tree of 1,778 *E. coli* ST405 genomes (See Figure 1) according to their color code
