# Supplementary material for: Comparative genomic analyses of Escherichia coli ST405 strains from Pakistan
Source: mSystems. 2026 Mar 16;11(4):e01685-25. doi: 10.1128/msystems.01685-25 (PMC13098264; doi:10.1128/msystems.01685-25)
Supplement: Fig. S2 — Abundance profiles of virulence factors in E. coli ST405 genomes. [file msystems.01685-25-s0002.docx]

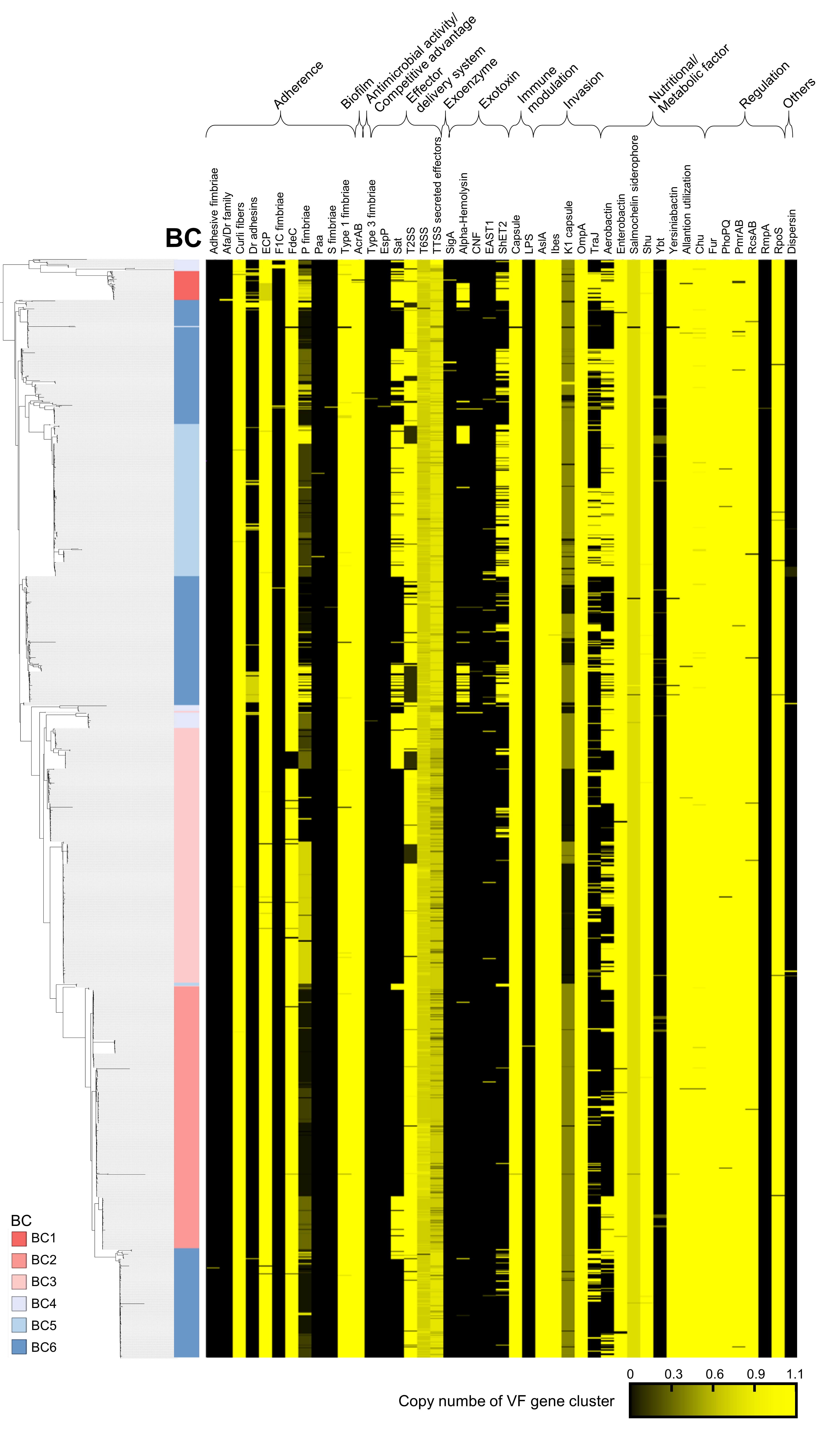


**FIG S2** Abundance profiles of virulence factors in *E. coli* ST405 genomes. Virulence factor gene cluster abundance profiles were incorporated into the phylogenomic tree of *E. coli* ST405 (see FIG 1). Complete conserved VF gene clusters were assigned a value of 1.0. Partially conserved clusters were quantified as the proportion of detected genes relative to the total number of genes in the cluster.
