# Supplementary material for: Comparative genomic analyses of Escherichia coli ST405 strains from Pakistan
Source: mSystems. 2026 Mar 16;11(4):e01685-25. doi: 10.1128/msystems.01685-25 (PMC13098264; doi:10.1128/msystems.01685-25)
Supplement: Fig. S4 — Plasmid replicon sites repertoires in BAPS clusters. [file msystems.01685-25-s0004.docx]

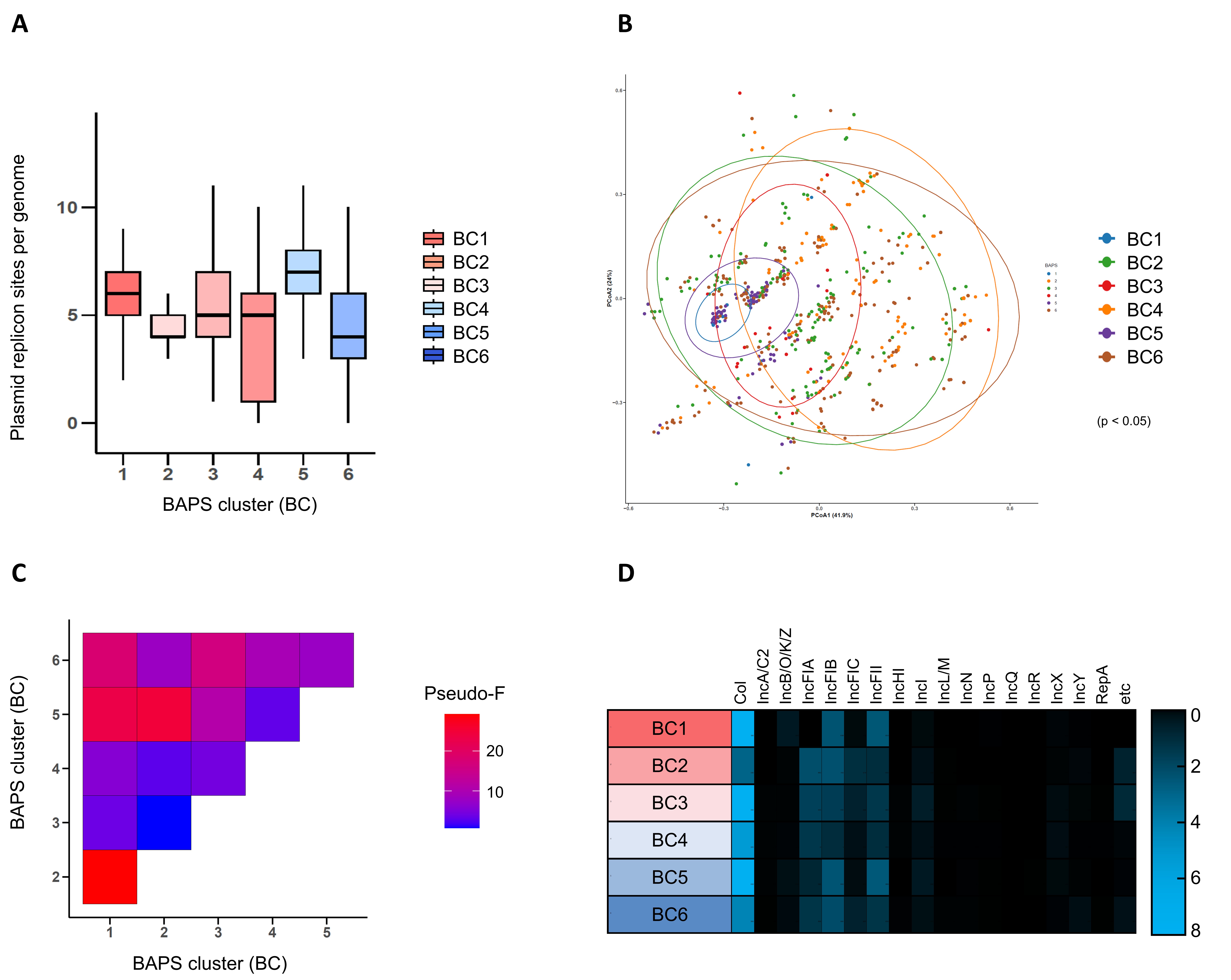


**FIG S4** Plasmid replicon sites repertoires in BAPS clusters (BCs). A, total amount of plasmid replicon sites in BAPS clusters; B, PCoA analyses using the Bray-Curtis difference matrix based on weighted plasmid replicon sites composition; C, statistical analyses of the difference between clusters using PERMANOVA; D, plasmid replicon site profiles in BCs displayed using site copy per population.
