# Supplementary material for: Comparative genomic analyses of Escherichia coli ST405 strains from Pakistan
Source: mSystems. 2026 Mar 16;11(4):e01685-25. doi: 10.1128/msystems.01685-25 (PMC13098264; doi:10.1128/msystems.01685-25)
Supplement: Fig. S5 — Sublineage-variable ARG repertoires in BAPS clusters. [file msystems.01685-25-s0005.docx]

**FIG S5** Sublineage-variable ARG repertoires in BAPS clusters (BCs). A, total amount of ARGs in BAPS clusters; B, PCoA analyses using the Bray-Curtis difference matrix based on weighted ARGs composition; C. statistical analyses of the difference between clusters using PERMANOVA; D, ARG profiles in BCs displayed using site copy per population.
