# Supplementary material for: Comparative genomic analyses of Escherichia coli ST405 strains from Pakistan
Source: mSystems. 2026 Mar 16;11(4):e01685-25. doi: 10.1128/msystems.01685-25 (PMC13098264; doi:10.1128/msystems.01685-25)
Supplement: Fig. S6 — Co-occurrence networks of ARGs, MGEs, and plasmid replicon types in ST405 subgroup A. [file msystems.01685-25-s0006.docx]

**
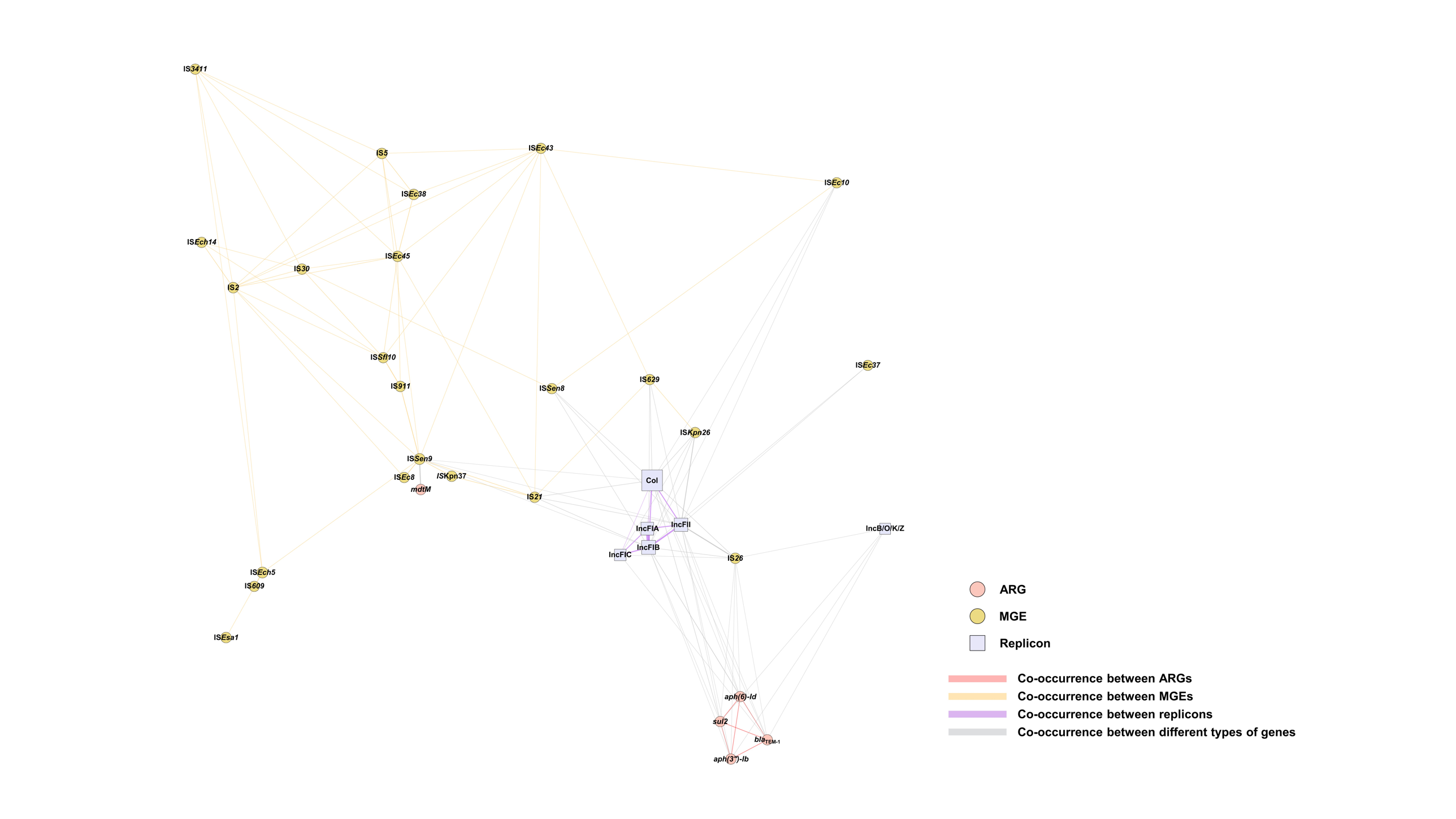
**

**FIG S6** Co-occurrence networks of ARGs, MGEs, and plasmid replicon types in ST405 subgroup A. Nodes represent ARGs (pink circles), MGEs (yellow circles), and plasmid replicon types (purple squares). Edges indicate pairs of genetic elements (ARG-ARG, ARG-MGE, and MGE-MGE) that co-occur within ±10 kb on the same contig, whereas ARG-replicon and MGE-replicon edges indicate ARGs or MGEs located on contigs carrying the corresponding plasmid replicon. Only pairs with a co-occurrence frequency of ≥1% within subgroup A are shown, and edge width is proportional to this frequency. Node positions were optimized using the ForceAtlas2 layout algorithm in Gephi.
