# Supplementary material for: Comparative genomic analyses of Escherichia coli ST405 strains from Pakistan
Source: mSystems. 2026 Mar 16;11(4):e01685-25. doi: 10.1128/msystems.01685-25 (PMC13098264; doi:10.1128/msystems.01685-25)
Supplement: Supplemental Tables — Tables S1 to S3, S5, and S6. [file msystems.01685-25-s0007.docx]

**Supplemental Tables**

**Table S1. Isolation information for *E. coli* strains isolated in Pakistan**

| Strain | Sample source | Sample type | Geographic location (longitude/latitude) | Collection date | Reference |
| --- | --- | --- | --- | --- | --- |
| PEC1013 | Bird | Feces | Shakarparian park, Islamabad  (33.6887°N 73.0887°E) | August 27, 2017 | This study |
| PEC1020 | Environment | Sewage water | H-sector Islamabad nullah  (33.6826° N 73.0649° E) | November 7, 2016 | This study |
| PEC1021 | Environment | Sewage water | Ministry of climate change Islamabad indoor  (33.7228° N 73.0962° E) | December 23, 2016 | This study |

**Table S2. Genome sequencing results of *E. coli* ST405 strains isolated in Pakistan**

| Strain name | Sequencing method | Status | Sequencing depth of coverage | N50 (bp) | Chromosome size (bp) | Plasmid  (size, bp) | GC  content (%) | No. of CDSs | No. of tRNA | No. of rRNA | NCBI GenBank accession No. |
| --- | --- | --- | --- | --- | --- | --- | --- | --- | --- | --- | --- |
| PEC  1013 | Sequel/  MiSeq | Complete | 561.02x | 5,124,833 | 5,121,833 | pPEC1013-1 (147,593)  pPEC1013-2 (94,194)  pPEC1013-3 (46,703)  pPEC1013-4 (1,459) | 50.7 | 5,046 | 90 | 22 | NZ_CP165737.1  NZ_CP165738.1  NZ_CP165739.1  NZ_CP165740.1  NZ_CP165741.1 |
| PEC  1020 | Sequel/  MiSeq | Complete | 743.43x | 5,167,906 | 5,167,906 | pPEC1020-1 (128,763)  pPEC1020-2 (48,552) | 50.6 | 4,963 | 87 | 22 | NZ_CP162603.1  NZ_CP162604.1  NZ_CP162605.1 |
| PEC  1021 | Sequel | Complete | 383.55x | 5,168,523 | 5,168,523 | pPEC1021-1 (93,230)  pPEC1021-2 (48,552)  pPEC1021-3 (128,762) | 50.6 | 5,073 | 90 | 22 | NZ_CP162406.1  NZ_CP162407.1  NZ_CP162408.1  NZ_CP162409.1 |

**Table S3. MLST analyses of 257,515 non-redundant *E. coli* genomes in the NCBI GenBank genome database (as of June 10, 2024)**

| ST | No. of genomes | Proportion  (%) | High-risk clone | Major *bla*_NDM-5_ carrier |
| --- | --- | --- | --- | --- |
| 11 | 29,965 | 11.6 |  |  |
| 131 | 16,955 | 6.6 | O |  |
| 21 | 13,242 | 5.1 |  |  |
| 17 | 11,606 | 4.5 |  |  |
| 10 | 10,794 | 4.2 | O |  |
| 16 | 6,179 | 2.4 |  |  |
| 69 | 3,961 | 1.5 | O |  |
| 58 | 3,464 | 1.3 | O |  |
| 73 | 3,379 | 1.3 | O |  |
| 38 | 3,226 | 1.3 | O |  |
| 95 | 2,765 | 1.1 | O |  |
| 410 | 2,692 | 1.0 | O | O |
| 117 | 2,654 | 1.0 | O |  |
| 32 | 2,653 | 1.0 |  |  |
| 155 | 2,479 | 1.0 |  |  |
| 167 | 2,346 | 0.9 | O | O |
| 101 | 2,205 | 0.9 | O |  |
| 655 | 2,175 | 0.8 |  |  |
| 1193 | 2,134 | 0.8 | O |  |
| 152 | 2,040 | 0.8 |  |  |
| 405 | 1,983 | 0.8 | O | O |
| Not assigned | 4,111 | 1.6 |  |  |
| Others | 124,507 | 48.3 |  |  |
| Total | 257,515 |  |  |  |

**Table S5. Sublineage-variable ARGs of *E. coli* ST405 genomes and their occurrence**

| Antibiotic class | ARG | No. of genomes carrying ARG | Abundance out of all 1,778 ST405 genomes  (%) | |
| --- | --- | --- | --- | --- |
| Aminoglycoside | *aac(3)-IIc* | 26 | 1.5 | |
|  | ***aac(3)-IId*** | **281** | **15.8** | |
|  | ***aac(3)-IIe*** | **355** | **20.0** | |
|  | *aac(6')-Ib10* | 44 | 2.5 | |
|  | *aadA* | 82 | 4.6 | |
|  | ***aadA2*** | **518** | **29.1** | |
|  | ***aadA5*** | **912** | **51.3** | |
|  | *aph(3')-Ia* | 61 | 3.4 | |
|  | ***aph(3'')-Ib*** | **605** | **34.0** | |
|  | ***aph(6)-Id*** | **662** | **37.2** | |
|  | *armA* | 24 | 1.3 | |
|  | ***rmtB*** | **301** | **16.9** | |
|  | ***aac(6')-Ib-cr6*** | **602** | **33.9** | |
| β-Lactam | *bla_CMY-2_* | 94 | 5.3 | |
|  | *bla_CMY-42_* | 128 | 7.2 | |
|  | *bla_CTX-M-14_* | 143 | 8.0 | |
|  | ***bla_CTX-M-15_*** | **1071** | **60.2** | |
|  | *bla_CTX-M-55_* | 36 | 2.0 | |
|  | *bla_KPC-2_* | 34 | 1.9 | |
|  | *bla_NDM-1_* | 37 | 2.1 | |
|  | ***bla_NDM-5_*** | **640** | **36.0** | |
|  | ***bla_OXA-1_*** | **641** | **36.1** | |
|  | *bla_OXA-48_* | 30 | 1.7 | |
|  | *bla_OXA-181_* | 26 | 1.5 | |
|  | ***bla_TEM-1_*** | **843** | **47.4** | |
| Disinfecting agents | ***qacEΔ1*** | **1377** | **77.4** | |
|  | *qacL* | 23 | 1.3 | |
| Sulfonamide | ***sul1*** | **1329** | **74.7** |  |
|  | ***sul2*** | **657** | **37.0** |  |
|  | *sul3* | 23 | 1.3 |  |
| Diaminopyrimidine | ***dfrA12*** | **507** | **28.5** |  |
|  | *dfrA14* | 62 | 3.5 |  |
|  | ***dfrA17*** | **865** | **48.7** |  |
|  | *dfrA24* | 30 | 1.7 |  |
| Fluoroquinolone | *mdtM* | 77 | 4.3 |  |
|  | *qepA2* | 45 | 2.5 |  |
|  | *qepA4* | 69 | 3.9 |  |
|  | *qnrS1* | 69 | 3.9 |  |
| Macrolide | ***ermB*** | **196** | **11.0** |  |
|  | ***mphA*** | **932** | **52.4** |  |
|  | *mphE* | 28 | 1.6 |  |
|  | ***mrx*** | **1184** | **66.6** |  |
|  | *msrE* | 28 | 1.6 |  |
|  | *tolC* | 140 | 7.9 |  |
| Glycopeptide | ***ble*(MBL)** | **658** | **37.0** |  |
| Phenicol | *catAl* | 86 | 4.8 |  |
|  | *catB3* | 29 | 1.6 |  |
|  | *cmlA1* | 15 | 0.8 |  |
|  | *cmlA5* | 19 | 1.1 |  |
|  | *cmlA6* | 33 | 1.9 |  |
|  | *floR* | 126 | 7.1 |  |
| Phosphonic acid | *fosA3* | 23 | 1.3 |  |
| Tetracycline | *emrK* | 33 | 1.9 |  |
|  | ***tet(A)*** | **693** | **39.0** |  |
|  | ***tet(B)*** | **845** | **47.5** |  |

*Blue letters indicate ARG exhibiting more than 10% abundance out of all ST405 genomes.

**Table S6. Information for pPEC1020-1, pPEC1021-3, and their homologous plasmids in the NCBI database (as of March 7, 2025).**

| Strain Name | Chromosome accession No. | Plasmid name | Plasmid  accession No. | Plasmid size  (bp) | Query coverage (%) | Identity  (%) | ST | Origin | Specimen | Isolation year | Country | ARG repertoires | Plasmid replicon types |
| --- | --- | --- | --- | --- | --- | --- | --- | --- | --- | --- | --- | --- | --- |
| PEC1021  (query sequence) | NZ_CP162406.1 | pPEC1021-3 | NZ_CP162409.1 | 128,762 | 100 | 100.00 | 405 | Sewage water |  | 2016 | Pakistan | *aadA2*, *dfrA12*, *mrx*, *bla*_NDM-5_, *qacE∆1*, *qepA4*, *sul1*, *bla*_TEM-1_, *tet(B)*, | IncFII |
| PEC1020 | NZ_CP162603.1 | pPEC1020-1 | NZ_CP162604.1 | 128,763 | 100 | 100.00 | 405 | Sewage water |  | 2016 | Pakistan | *aadA2*, *dfrA12*, *mrx*, *bla*_NDM-5_, *qacE∆1*, *sul1*, *bla*_TEM-1_, *tet(B)* | IncFIB, IncFII |
| 98201 | NZ_CP173561.1 | p98201-contig_1 | NZ_CP173563.1 | 128,763 | 100 | 100.00 | 156 | Homo sapiens | Urine | 2020/  2023 | Egypt | *bla*_CTX-M-15_, *dfrA12*, *mrx*, *bla*_NDM-5_,  *qacE∆1*, *qepA4*, *sul1*, *bla*_TEM-1_, *tet(B)* | IncFIB, IncFII |
| FDAARGOS_448 | NZ_CP023960.1 | plasmid 1 | CP023959.1 | 128,761 | 100 | 100.00 | 405 | Homo sapiens | Urine | 2014 | Canada | *aadA2*, *bla*_CTX-M-15_, *dfrA12*, *mphA*, *mrx*, *bla*_NDM-5_, *qacE∆1*, *qepA4*, *sul1*, *bla*_TEM-1_, *tet(B)* | IncFIB, IncFII |
| 15978 |  | pHN15978-1 |  | 128,762 | 100 | 100.00 | ND | Retail meat |  |  | Pakistan | *aadA2*, *dfrA12*, *mphA*, *mrx*, *bla*_NDM-5_,  *qacE∆1*, *qepA2*, *sul1*, *bla*_TEM-1_, *tet(B)* | IncFIB, IncFII |
| Ec355340 |  | pEc355340_NDM-5 |  | 128,841 | 100 | 99.99 | ND |  |  |  |  | *aadA2*, *dfrA12*, *mphA*, *mrx*, *bla*_NDM-5_,  *qacE∆1*, *qepA4*, *sul1*, *bla*_TEM-1_, *tet(B)* | IncFIB, IncFII |
| dm925c | JAGIKE000000000.1 | p_dm925c_NDM5 | NZ_CP095665.1 | 123,766 | 96 | 99.99 | 405 | Homo sapiens | Urine | 2017 | Bangladesh | *aadA2*, *dfrA12*, *mphA*, *mrx*, *bla*_NDM-5_,  *qacE∆1*, *qepA4*, *sul1*, *bla*_TEM-1_, *tet(B)* | IncFII |
| AR_452 | NZ_CP030331.1 | plasmid 1 | NZ_CP030329.1 | 128,762 | 100 | 100.00 | 156 |  |  |  |  | *aadA2*, *dfrA12*, *mphA*, *mrx*, *bla*_NDM-5_,  *qacE∆1*, *qepA4*, *sul1*, *bla*_TEM-1_, *tet(B)* | IncFIB, IncFII |
| Survcare321 | NZ_CP076301.1 | p321-NDM5 | NZ_CP076302.1 | 127,528 | 99 | 99.99 | 405 | Homo sapiens | Urine | 2019 | Germany | *aadA2*, *dfrA12*, *mphA*, *mrx*, *bla*_NDM-5_,  *qacE∆1*, *qepA4*, *sul1*, *bla*_TEM-1_, *tet(B)* | IncFII |
| 98207 | NZ_CP173539.1 | p98207-contig_3 | NZ_CP173538.1 | 129,599 | 100 | 99.99 | 156 | Homo sapiens | swab | 2020/  2023 | Egypt | *aadA2*, *dfrA12*, *mphA*, *mrx*, *bla*_NDM-5_,  *qacE∆1*, *qepA4*, *sul1*, *bla*_TEM-1_, *tet(B)* | IncFIB, IncFII |
| 98221 | NZ_CP173484.1 | p98221-contig_1 | NZ_CP173483.1 | 129,539 | 100 | 100.00 | 156 | Homo sapiens | Urine | 2020/  2023 | Egypt | *aadA2*, *bla*_CTX-M-15_, *dfrA12*, *mphA*, *mrx*, *bla*_NDM-5_, *qacE∆1*, *qepA4*, *sul1*, *bla*_TEM-1_, *tet(B)* | IncFIB, IncFII |
| KY 1497 | NZ_AP019803.1 | pKY1497_1 | NZ_AP019804.1 | 123,767 | 96 | 100.00 | 405 | Homo sapiens | Urine | 2015 | Japan | *aadA2*, *dfrA12*, *mphA*, *mrx*, *bla*_NDM-5_,  *qacE∆1*, *qepA4*, *sul1*, *bla*_TEM-1_, *tet(B)* | IncFIB, IncFII |
| EC190329 | NZ_CP142949.1 | pEC190329_1 | NZ_CP142950.1 | 129,346 | 98 | 99.96 | 405 | Homo sapiens | Rectal swab | 2019 | Ireland | *aadA2*, *dfrA12*, *mphA*, *mrx*, *bla*_NDM-5_,  *qacE∆1*, *qepA4*, *sul1*, *bla*_TEM-1_, *tet(B)* | IncFII |
| 98234 | NZ_CP173442.1 | p98234-contig_4 | NZ_CP173441.1 | 128,762 | 100 | 100.00 | 156 | Homo sapiens | Urine | 2020/  2023 | Egypt | *aadA2*, *aadA5*, *bla*_CTX-M-15_, *dfrA12*, *dfrA17*, *ermB*, *mphA*, *mrx*, *bla*_NDM-5_, *qacE∆1*, *sul1*, *bla*_TEM-1_, *tet(B)* | IncFIB, IncFII |
| dm874 | JAGIJP000000000.1 | p_dm874_NDM5 | NZ_CP095659.1 | 128,787 | 100 | 99.87 | 405 | Homo sapiens | Urine | 2017 | Bangladesh | *aadA2*, *dfrA12*, *mrx*, *bla*_NDM-5_, *qacE∆1*, *qepA4*, *sul1*, *bla*_TEM-1_, *tet(B)* | IncFII |
| 98191 | NZ_CP173600.1 | p98191-contig_2 | NZ_CP173599.1 | 128,762 | 100 | 99.99 | 156 | Homo sapiens | Urine | 2020/  2023 | Egypt | *aadA2*, *dfrA12*, *mrx*, *bla*_NDM-5_,  *qacE∆1*, *sul1*, *bla*_TEM-1_, *tet(B)* | IncFIB, IncFII |
| 2021CK-00607 | NZ_CP104665.1 | plasmid 1 | NZ_CP104666.1 | 131,846 | 95 | 100.00 | 648 | Homo sapiens | Urine | 2021 | USA | *aadA2*, *dfrA12*, *mrx*, *bla*_NDM-5_, *qacE∆1*, *qepA4*, *sul1*, *bla*_TEM-1_, *tet(B)* | IncFII |
| FUJ80154 | NZ_AP024687.1 | pFUJ80154-1 | NZ_AP024688.1 | 137,114 | 95 | 99.99 | 648 | Homo sapiens |  | 2019 | Japan | *aadA2*, *dfrA12*, *mphA*, *mrx*, *bla*_NDM-5_,  *qacE∆1*, *qepA4*, *sul1*, *bla*_TEM-1_, *tet(B)* | IncFII |
| M2021_10044824  _1_E | NZ_CP163448.1 | p10044824_1 | NZ_CP163449.1 | 132,040 | 99 | 99.83 | 405 | Pork |  | 2021 | Hungary | *aadA2*, *aadA5*, *dfrA12*, *dfrA17*, *ermB*, *mphA*, *mrx*, *bla*_NDM-5_, *qacE∆1*, *sul1*, *bla*_TEM-1_, *tet(B)* | IncFIB, IncFII |
| M2021-10044802/2-E | NZ_CP163451.1 | p10044802_1 | NZ_CP163453.1 | 131,993 | 99 | 99.82 | 405 | Cattle |  | 2021 | Hungary | *aadA2*, *dfrA12*, *mphA*, *mrx*, *bla*_NDM-5_,  *qacE∆1*, *sul1*, *bla*_TEM-1_, *tet(B)* | IncFIB, IncFII |
| GENOMIC22-003 | CP119116.1 | pCFSAN126948_02 | CP119118.1 | 132,041 | 99 | 100.00 | 405 | Environmental  /food/other |  | 2022 | Denmark | *aadA2*, *dfrA12*, *mphA*, *mrx*, *bla*_NDM-5_,  *qacE∆1*, *qepA4*, *sul1*, *bla*_TEM-1_, *tet(B)* | IncFIB, IncFII |

*Shades indicate plasmids from ST405 strains.
